# Characterization of AnNce102 and its role in eisosome stability and sphingolipid biosynthesis

**DOI:** 10.1038/srep15200

**Published:** 2015-10-15

**Authors:** Alexandros Athanasopoulos, Christos Gournas, Sotiris Amillis, Vicky Sophianopoulou

**Affiliations:** 1 Institute of Biosciences and Applications, Microbial Molecular Genetics Laboratory, National Center for Scientific Research, Demokritos (NCSRD), Athens, Greece; 2 Faculty of Biology, University of Athens, Panepistimioupolis 15781, Athens, Greece

## Abstract

The plasma membrane is implicated in a variety of functions, whose coordination necessitates highly dynamic organization of its constituents into domains of distinct protein and lipid composition. Eisosomes, at least partially, mediate this lateral plasma membrane compartmentalization. In this work, we show that the Nce102 homologue of *Aspergillus nidulans* colocalizes with eisosomes and plays a crucial role in density/number of PilA/SurG foci in the head of germlings. In addition we demonstrate that AnNce102 and PilA negatively regulate sphingolipid biosynthesis, since their deletions partially suppress the thermosensitivity of *basA* mutant encoding sphingolipid C4-hydroxylase and the growth defects observed upon treatment with inhibitors of sphingolipid biosynthesis, myriocin and Aureobasidin A. Moreover, we show that YpkA repression mimics genetic or pharmacological depletion of sphingolipids, conditions that induce the production of Reactive Oxygen Species (ROS), and can be partially overcome by deletion of *pilA* and/or *annce102* at high temperatures. Consistent with these findings, *pilA*Δ and *annce102*Δ also show differential sensitivity to various oxidative agents, while AnNce102 overexpression can bypass sphingolipid depletion regarding the PilA/SurG foci number and organization, also leading to the mislocalization of PilA to septa.

The plasma membrane (PM) is involved in a variety of functions such as cell adhesion, endocytosis, exocytosis, while serving as a platform for various signalling complexes. In the last years, it has increasingly become evident that the PM is highly compartmentalized for accomplishing these roles^1^,^2^. Studies mainly with *Saccharomyces cerevisiae* and other ascomycetes focused on the lateral organization of biological membranes, have shown that PM is organized into numerous partially overlapping domains^3^, some of which are protein organized and large enough to be monitored by fluorescence microscopy. But how these domains arise is poorly understood, largely due to the technical difficulties in studying hydrophobic membranes^4^.

In the PM of yeast, three types of distinct spatial domains with different lipid and protein composition are well characterized: MCP, MCC and MCT. The first identified domain (Membrane Compartment of Pma1p, MCP) was found to contain the highly expressed plasma membrane H^+^-ATPase - Pma1p, which forms a network-like pattern^5^. In contrast to MCP, MCC (Membrane Compartment of Can1p, arginine permease) appears as large immobile patches of roughly 300 nm diameter forming long furrow-like invaginations that contain several transmembrane proteins^6^,^7^ and members of the Sur7 and Nce102 families of tetraspan proteins^8^,^9^. MCC organization is at least in part mediated by the Nce102 protein^10^ and by a cellular stable structure termed eisosome, lying underneath MCC. Each eisosome is composed of three proteins in thousands of copies, the phylogenetically related cytoplasmic Pil1 and Lsp1 and the transmembrane Sur7 protein^11^. Eisosomes in *S. cerevisiae* are organized/regulated by the phosphorylation of Pil1 and Lsp1 by the Pkh1/2 kinase (homologues of mammalian 3-phosphoinositide-dependent kinase) and the levels of sphingolipid Long-Chain Bases (LCBs)^12^,^13^,^14^. Sphingolipids, especially abundant complex sphingolipids, are important structural components of eukaryotic cell membranes. In addition to their structural roles, sphingolipid metabolites such as ceramides and LCBs can act as signaling molecules in many cellular processes, including cell migration, stress response, survival, apoptosis, senescence, differentiation and endocytosis^15^,^16^. The tetraspan protein Nce102 has been implicated as part of a sensor for sphingolipid homeostasis^10^. Membrane stress, including sphingolipid depletion, triggers eisosome proteins Slm1/2, to move out of MCC and associate with the third well characterized PM domain, MCT, Membrane Compartment of TORC2 (target of rapamycin kinase complex 2)^10^,^17^. The Slm1/2 proteins then recruit Ypk1 to the PM, where it is phosphorylated by Tor2 and Pkh1/2, in order to be fully activated^17^,^18^. Once fully activated, Ypk1 phosphorylates and consequently inactivates the endoplasmic reticulum (ER)-localized proteins, Orm1 and Orm2^17^,^19^,^20^. Furthermore, it stimulates the function of the ceramide synthase complex, by increasing the rate of the formation of ceramides and preventing hyper-accumulation of LCBs/LCBPs, thus avoiding inadvertent induction of autophagy under sufficient conditions^21^.

In the model filamentous fungus *Aspergillus nidulans*, PilA, PilB and SurG, homologues of the *S. cerevisiae* eisosome proteins Pil1/Lsp1 and Sur7, are assembled and form tightly packed structures^22^. In conidiospores and ascospores, the three proteins colocalize at the cell cortex forming stable structures that differ from the clearly distinct eisosome patches observed in *S. cerevisiae*^22^,^23^. The aim of the present study was to use the genetically tractable filamentous fungus *Aspergillus nidulans* for a detailed live-cell imaging and characterization of AnNce102 at different developmental stages of fungus asexual life cycle. We examined the contribution of AnNce102 to the organization/stability of eisosome foci and its possible role in sphingolipid biosynthesis and YpkA (YPK1 homologue) signaling. Our results indicate that AnNce102 colocalizes with eisosomes and affects the density/number of PilA/SurG foci in the head of germlings. Myriocin treatment, similar to *annce102Δ*, results in PilA foci disassembly, a phenotype that can be overcome by overexpression of AnNce102. In addition we show that the main organizers of eisosomes, the PilA and AnNce102 proteins, negatively regulate sphingolipid biosynthesis.

## Results

### Nce102 of *A. nidulans* is an eisosomal protein

**Figure 1:**
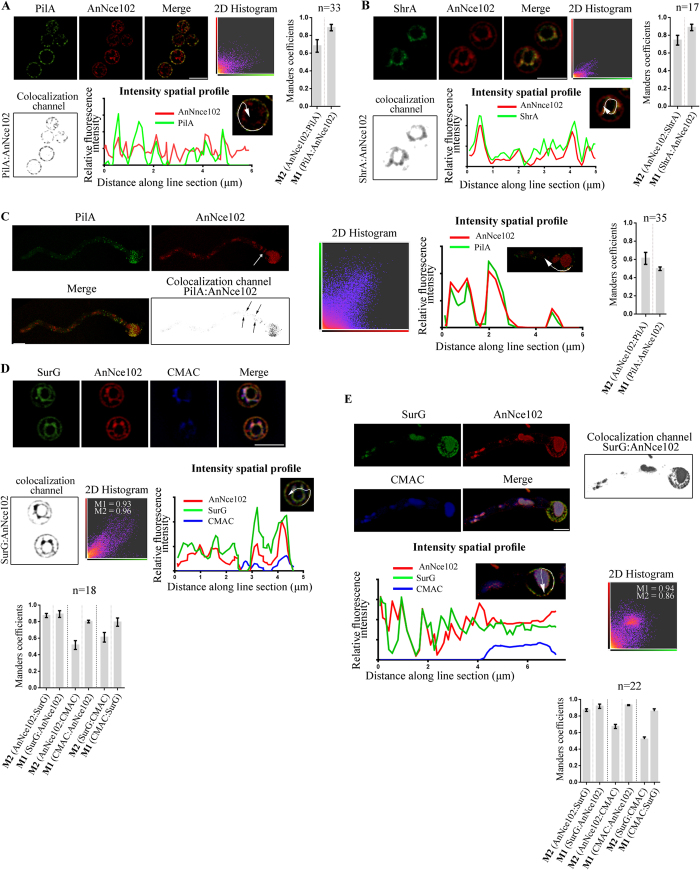
AnNce102 colocalizes with PilA and SurG eisosomal proteins. Deconvoluted confocal images and colocalization analysis (see materials and methods) of AnNce102/PilA (**A**) and AnNce102/ShrA (**B**) in quiescent conidia, AnNce102/PilA in germlings (**C**) and AnNce102/SurG in quiescent conidia (**D**) and germlings (**E**). Every dataset (**A**,**B**,**C**,**D**,**E**) contains a 2D histogram obtained using Imaris. Colocalization was represented as new channel (colocalization channel as inverted gray scale images in black and white) corresponding to the colocalized voxels. Histograms show Manders colocalization coefficients (M1 and M2), with averages presented as mean ± SEM, where n represents the number of cells examined from at least three independent experiments. Analysis of fluorescent intensities (intensity spatial profile) along line section is shown for every dataset. A, B and D represent equatorial sections of confocal stacks and C and E represent volumetric views of confocal stacks generated by Imaris. Scale Bars 5 μm.

**Figure 2:**
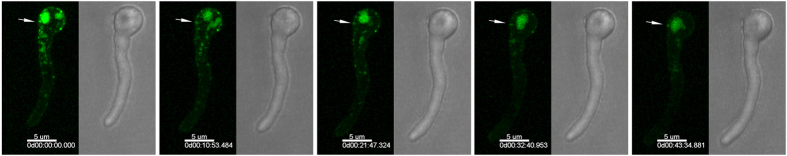
AnNce102 forms stable structures with low mobility. Images were acquired every 150 seconds. White arrow indicates representative AnNce102 spot that remains stable more than 39 minutes. Scale Bars 5 μm.

The *S. cerevisiae* Nce102 sequence (YPR149W) was used as template to identify homologues in the *A. nidulans* genome using BlastP. The top-scoring match corresponds to AN7683 Open Reading Frame (NCBI-GeneID:2869016) of 633 bp encoding an 174 amino acid protein, characterized by the presence of a MARVEL (Myelin And Lymphocyte and Related Proteins for Vesicle Trafficking and Membrane Link) domain (PFAM domain PF01284; http://www.sanger.ac.uk/Software/Pfam/index.shtml). To investigate the intracellular localization of AnNce102, C-terminal fusions of *annce102* open reading frame with GFP and mRFP fluorophores were constructed, expressing *annce102* from its endogenous promoter. Deconvoluted Z stacks of AnNce102 in quiescent conidia showed a plasma membrane staining pattern that colocalizes with PilA and partially localizes in the remainder of the membrane (Fig. 1A). AnNce102 localizes additionally in intracellular structures resembling the Endoplasmatic Reticulum (ER), as shown by its colocalization with the ER chaperone, ShrA^24^ (Fig. 1B). During isotropic growth, the AnNce102 ER fluorescence signal diminishes and it disappears before the emergence of the germination tube (Fig. 1C and below). In germlings, AnNce102 foci are mostly confined to eisosomes of the hyphal head and to vacuoles, as stainable with the vacuolar tracer, CMAC. Additionally, AnNce102 is clearly detected in septa of germlings (Fig. 1C, white arrow) and colocalizes with some PilA foci along the hyphal plasma membrane (Fig. 1C, black arrows). This temporal and spatial AnNce102 distribution resembles that of the SurG tetraspan eisosomal protein (Fig. 1D,E)^22^. Moreover using time-lapse live-cell imaging, we show that AnNce102 forms stable structures with low mobility at fungal plasma membrane (Fig. 2).

### Deletion of *pilA* leads to low levels of AnNce102 and SurG in growing germlings

**Figure 3:**
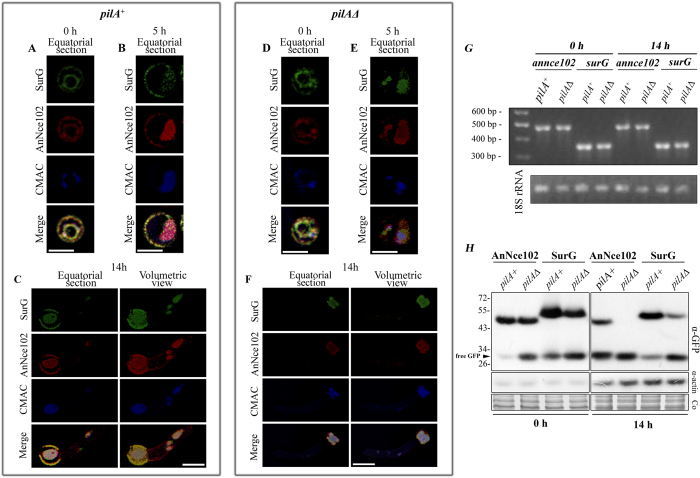
Deletion of *pilA* leads to enhanced degradation of AnNce102 and SurG. Deconvoluted confocal images of AnNce102-mRFP and SurG-GFP in quiescent (0 h) and swollen (5 h) conidia and germlings (14 h) of (**A**–**C**) *pilA*^+^ and (**D**–**F**) *pilA*Δ strains, respectively, stained with the vacuolar tracer, CMAC. Scale Bars 5 μm. (**G**) Expression of *annce102* and *surG* genes in quiescent conidia (0 h) and germlings (14 h) of *pilA*^+^ and *pilA*Δ strains using semi-quantitative RT-PCRs. (**H**) Western blot analysis of AnNce102-GFP and SurG-GFP proteins derived from quiescent conidia (0 h) and young hyphae (14 h) of *pilA*^+^ and *pilA*Δ strains. Detection of actin and Coomassie staining (Co) are also shown as loading controls. Notice the lower signal of actin in the samples from quiescent conidia, which is due to the developmental expression of actin (*acnA*) during conidiospore germination^59^.

In yeast cells, deletion of *PIL1* disrupts MCCs and all remaining eisosome proteins investigated so far coalesce into one or a few punctate foci at the cell periphery, referred to as eisosome remnants^7^,^13^. In addition, Sur7 and Nce102 localize to a few bright clusters and a pool diffusely distributed over the plasma membrane^10^,^13^. On the contrary, deletion of *pilA* in quiescent conidia of *A. nidulans* has a minor effect on AnNce102 and SurG distribution. Both proteins appear to be localized less on the membrane and more in vacuoles compared to wild type cells (Fig. 3A,D). Similarly, almost no fluorescence is detected on the membrane during isotropic growth (Fig. 3E), while in germlings both proteins are localized exclusively in vacuoles and endosomes (Fig. 2F). RT-PCRs in total RNA extracted from quiescent conidia (0 h) and germlings (14 h) of a wild type strain and *pilA*Δ revealed that mRNAs of both *annce102* and *surG* are abundant throughout the time-interval tested without significant differences (Fig. 3G). In contrast, western blot, using protein extracts from quiescent conidia (0 h) and germlings (14 h) confirmed microscopic observations. More specifically, deletion of *pilA* causes enhanced degradation of AnNce102-GFP and SurG-GFP in the vacuoles, as evidenced by the diminished signal of intact GFP-tagged proteins and the increased quantity of free GFP, even in quiescent conidia (Fig. 3H). We should also mention that deletion of *pilB* did not affect the membrane localization of AnNce102 and SurG of young germlings, while *pilA*Δ *pilB*Δ double mutants did not show any difference from *pilA*Δ single mutants (Supplementary Fig. S1). Together, these data suggest that normal eisosomal organization requires PilA.

### Deletion of *annce102* leads to fewer PilA and SurG foci in the head of germlings

**Figure 4:**
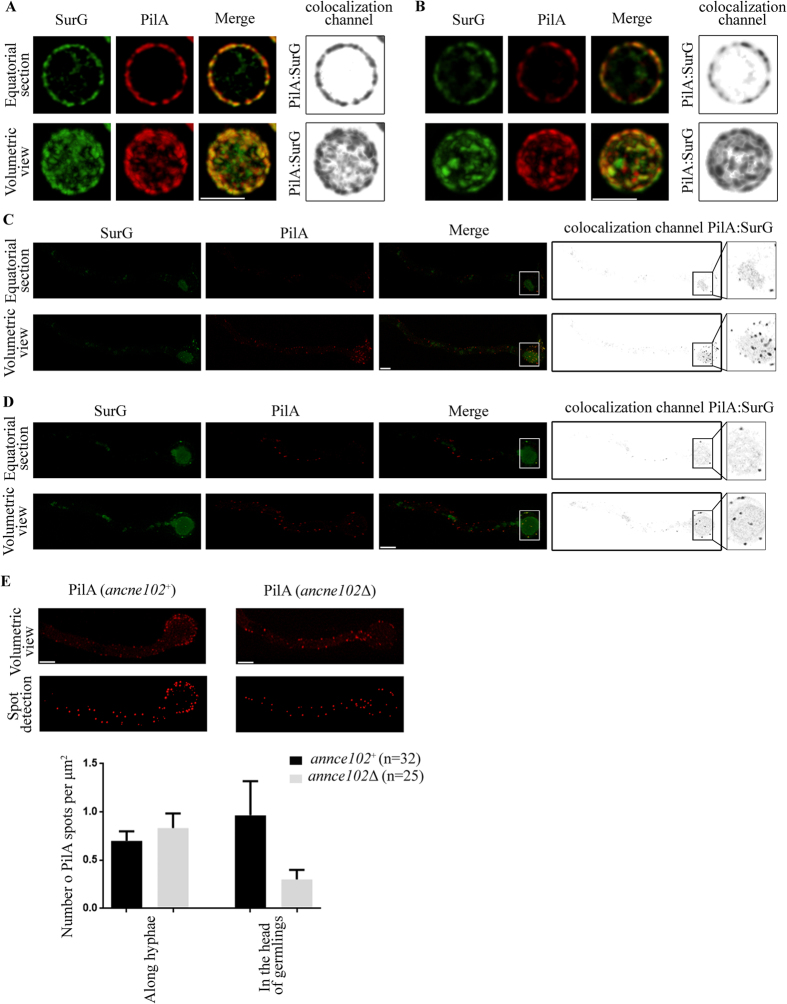
Deletion of *annce102* leads to fewer PilA and SurG foci in the head of germlings. Deconvoluted confocal images of PilA-mRFP and SurG-GFP in quiescent conidia (0 h) and germlings (14 h) of *annce102*^+^ (**A**,**C**) and *annce102*Δ (**B**,**D**) strains, respectively. (**E**) The number of PilA foci in *annce102*^+^ and *annce102*Δ cells was counted using the spot tool of the Imaris software and is shown below the images, as a graph with averages presented as mean ± SEM, where n represents the number of cells examined from at least three experiments. Scale Bars 3 μm.

**Figure 5:**
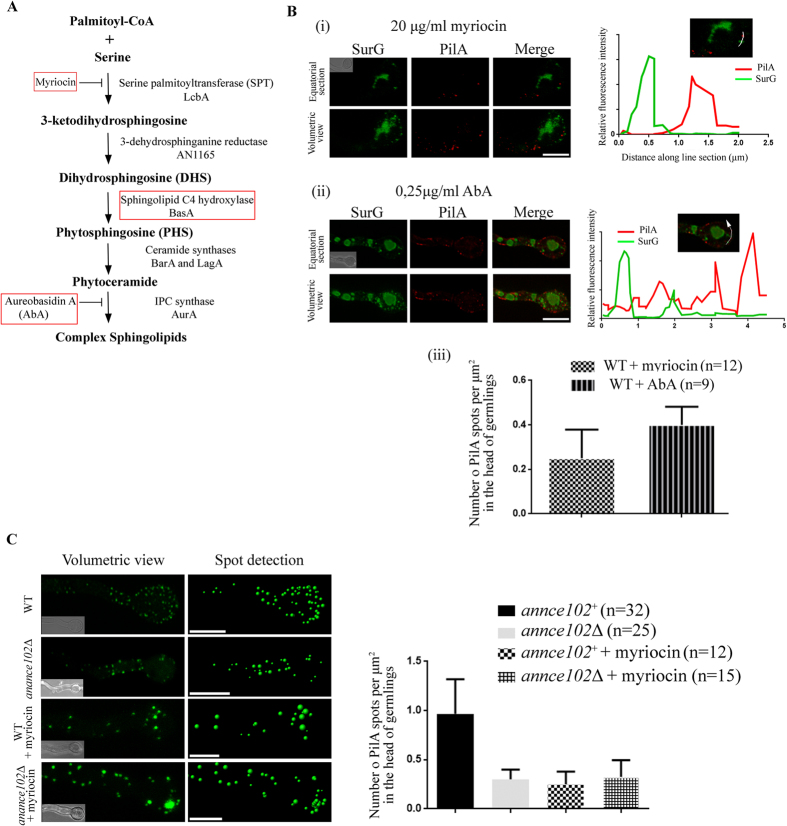
(**A**) A simplified scheme of the sphingolipid synthesis pathway of *A. nidulans*^29^. Genes and inhibitors used in this study are shown in red boxes. (**B**) Deconvoluted confocal images of PilA-mRFP and SurG-GFP, shown as equatorial sections and volumetric view of confocal stacks, treated for 5 h with 20 μg/ml myriocin (**i**) and 0,25 μg/ml Aureobasidin A (AbA) (**ii**). The number of PilA foci was counted using the spot tool of the Imaris software and is shown as a graph with averages presented as mean ± SEM, where n represents the number of cells examined from at least three independent experiments (**iii**) (**C**) Representative deconvoluted confocal images of wild type and *annce102*Δ cells expressing PilA-GFP, treated for 5 h with 20 μg/ml myriocin. The number of PilA foci was counted as above and is shown as a graph with averages presented as mean ± SEM, where n represents the number of cells examined from at least three independent experiments. Scale Bars 5 μm.

*annce102* deletion mutants displayed normal growth at 25, 30, 37, and 42 °C (see Materials and Methods), while conidia exhibited properties of swelling and polarity establishment (time of germination tube appearance) indistinguishable from that of a wild type strain at all temperatures tested (Supplementary Fig. S2). Contrary to *S. cerevisiae*, where *nce102*Δ mutants displayed altered localization of Sur7-GFP, Pil1-GFP, Can1-GFP, and ergosterol^8^,^10^, deletion of the *annce102* gene has no significant effect on PilA-mRFP and SurG-GFP distribution in quiescent conidia of *A. nidulans*, with both proteins colocalizing on the membrane (Fig. 4A,B). In *annce102Δ* germlings however, PilA and SurG displayed an uneven distribution compared to wild type (Fig. 4C,D). Using the spot tool of the Imaris software, we found that PilA forms 3-fold fewer foci in the head of *annce102Δ* germlings (Fig. 4E). Interestingly, a similar effect is observed in the presence of sub-lethal doses of myriocin (Fig. 5A) (inhibitor of serine palmitoyltransferase, the enzyme catalyzing the first step in sphingolipid biosynthesis), where in addition no colocalization of PilA-mRFP and SurG-GFP foci is observed (Fig. 5Bi). The similarity of the phenotypes suggests that, like in yeast^10^, myriocin could affect the organization of eisosomes through AnNce102. To add to the significance of the above, the effects of myriocin addition and *annce102* deletion on the number of PilA foci were not additive (Fig. 5C), while overexpression of *annce102* can protect from myriocin-induced eisosome disassembly (see below).

The myriocin effect on eisosome localization may be due to the lack of LCBs, complex sphingolipids or a more general response to sphingolipids overall. To distinguish between these possibilities, we tested the effect of Aureobasidin A (AbA), a specific inhibitor of complex sphingolipids^25^ (Fig. 5A). In PilA-mRFP/SurG-GFP cells treated with AbA, although we observed less pronounced reduction of PilA foci in the head of conidia-derived germlings, the treated cells displayed enhanced degradation of SurG (Fig. 5 Bii and Biii, compare with 4E), suggesting that proper eisosomal organization depends on the overall level of sphingolipids. Together, the above data show that normal eisosomal organization requires AnNce102 and suggests that this protein is involved in sphingolipid biosynthesis.

Having shown that the AnNce102 protein affects eisosomal foci number in the head of germlings, and given that PilA is the only eisosomal protein displaying a punctate distribution pattern along the hypha^22^, we attempted to identify other proteins that could potentially affect the distribution/number of PilA foci along hypha. We chose to study proteins that exhibit high identity to the amino acid sequence of AnNce102 MARVEL-containing protein. *In silico* analysis of the genome of *A. nidulans* revealed 2 sequences, AN8278 and AN8422, designated *mrvA* and *mrvB*, showing 26.9% and 23.9% identity to AnNce102 and displaying 9.0e-07 and 6.0e-03 E values to AnNce102, respectively. However, strains with deletions in one or both genes exhibited normal growth in all conditions tested and no changes in the topology of PilA in both conidia and germlings were observed (Supplementary Fig. S3). These results suggest that *mrvA* and *mrvB* do not participate in eisosome stability/organization.

### Overexpression of AnNce102 restores PilA foci number in the presence of myriocin and leads to ectopic localization of PilA foci into septa

**Figure 6:**
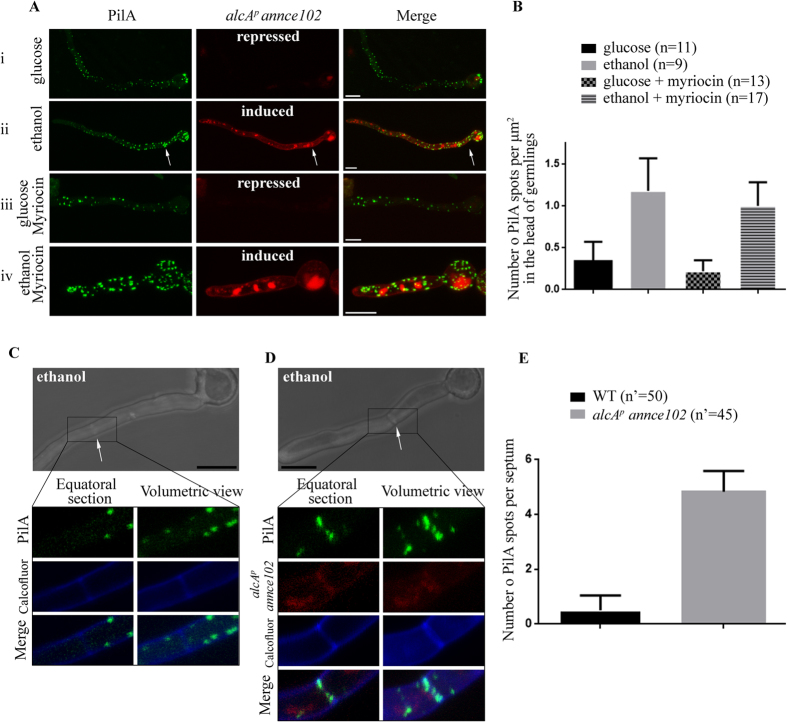
Overexpression of AnNce102 suppresses myriocin-induced eisosome disassembly and causes mislocalization of PilA foci to septa. Deconvoluted confocal images of WT (PilA-GFP) and *alcA*^*p*^ *annnce102* (*alcA*^*p*^ AnNce102-mRFP) strains grown in the presence of (**Ai**) glucose, (**Aii**) ethanol, (**Aiii**) glucose and 20 μg/ml myriocin and (**Avi**) ethanol and 20 μg/ml myriocin. (**B**) The number of PilA foci every case was counted using the spot tool of the Imaris software and is shown as a graph. Representative confocal images of (**C**) PilA-GFP and (**D**) PilA-GFP *alcA*^*p*^-AnNce102-mRFP strains, in the presence of ethanol as sole carbon source. Septa are visualized by calcofluor white staining and marked with white arrows. Insets show a higher magnification of the boxed area. (**E**) The number of PilA foci per septum was counted and is shown as a graph with averages presented as mean ± SEM, where n represents the number of cells examined from at least three independent experiments. Bars 5 μm.

In order to test the effect of *annce102* overexpression on PilA foci number in myriocin-treated cells, a conditional *annce102* mutant strain was constructed, *alcA::annce102::mRFP*, expressing *annce102* under the control of the alcohol dehydrogenase (*alcA*) promoter, which is repressed by glucose and induced by ethanol^26^. Under repressing conditions the number of PilA foci in the head of germlings is similar to that of *annce102*Δ cells (Fig. 6Ai). Under inducing conditions, AnΝce102 is diffusely distributed to the periphery of cells, although some colocalization with PilA foci can be detected in the head of germlings. Most importantly, overexpression of AnNce102 in the presence of myriocin results in normal PilA foci number in the head of germlings (Fig. 6B).

Interestingly, under conditions of induced expression of AnNce102, PilA foci are also clearly localized to septa (Fig. 6, white arrows). As shown in Fig. 6C (white arrows), PilA foci do not localize to calcofluor white-stained septa of *A. nidulans*. However, overexpression of AnNce102 causes mislocalization of PilA foci to septa without however, affecting their morphology. These data confirm the involvement of AnNce102 in sphingolipid sensing and demonstrate the crucial role of AnNce102 regarding PilA foci number and topology.

### Sphingolipid depletion and *ypkA* repression increase ROS levels

**Figure 7:**
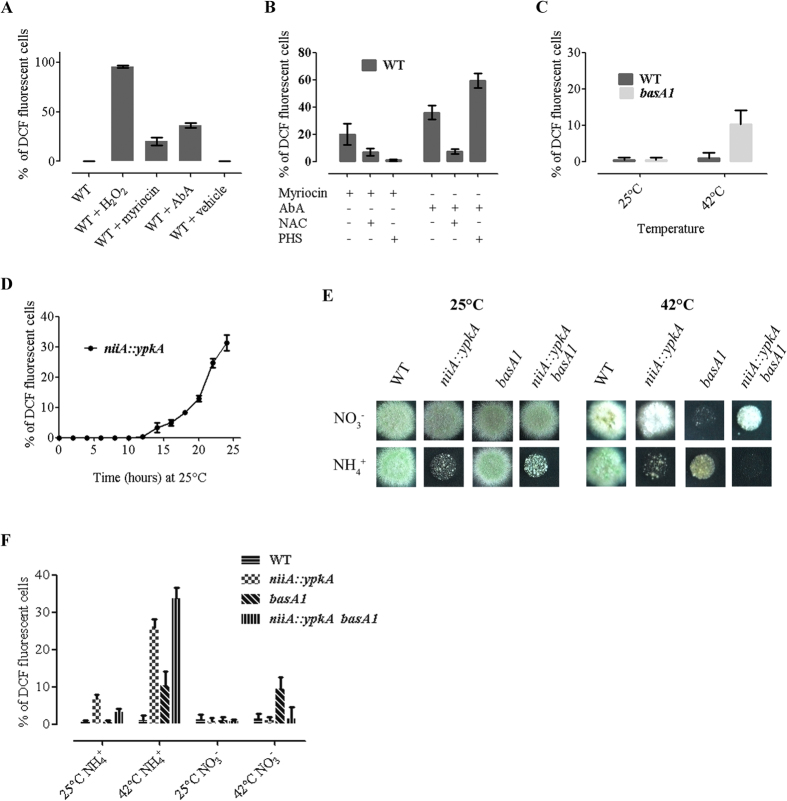
ROS accumulation by sphingolipid depletion and *ypkA* repression. (**A**) WT cells grown in MM for 12 h at 25 °C and then treated for 7h with 0,5 mM H_2_O_2_, 40 μg/ml myriocin, 2 μg/ml AbA and methanol (vehicle). All strains were incubated with 10 mM 2, 7 -dichlorofluorescin diacetate (DCF) for the last 45 min prior to imaging by fluorescence microscopy. Quantification represents percentage of 200–500 cells labeled with DCF, from at least three representative independent experiments, with averages presented as mean ± SEM. (**B**) WT cells grown in MM for 12 h at 25 °C, and then treated for 7 h with 40 μg/ml myriocin, 2 μg/ml AbA, 20 mM NAC and 2 μg/ml PHS. ROS were determined and quantified as in (**A**). (**C**) WT and *basA1* cells grown in MM for 12 h at 25 °C and shifted at 42 °C for additional 7 h. ROS were determined and quantified as in (**A**). (**D**) *niiA::ypkA* cells grown in MM for 0–24 h at 25 °C under repressing (NH_4_^+^) conditions. ROS were determined and quantified every 2 h intervals as in (**A**). (**E**) 5 μl of 10^7^/ml conidia of WT, *niiA::ypkA, basA1* and *niiA::ypkA basA1* cells spotted on MM and grown for 48 h at 25 °C or 42 °C, under repressing or inducing conditions. (**F**) WT, *niiA::ypkA, basA1* and *niiA::ypkA basA1* cells grown in MM for 12 h at 25 °C, under repressing or inducing conditions. Strains remain at 25 °C or shifted at 42 °C for additional 7 h, as indicated. ROS were determined and quantified as in (**A**).

In *S. cerevisiae* it has been shown that perturbation of sphingolipid metabolism leads to loss of cell viability and Reactive Oxygen Species (ROS) accumulation^16^. In addition, deletion of *nce102* confers higher sensitivity to oxidative stress^27^. Thus, we have investigated whether sphingolipid depletion regulates ROS accumulation in *A. nidulans* as well. Toward this end, we studied cells treated with myriocin and Aureobasidin A, specific inhibitors of sphingolipid biosynthesis^25^,^28^, as well as cells genetically blocked for sphingolipid biosynthesis. 20% of myriocin and 36% of AbA treated cells displayed increased ROS levels, visualized by the ROS indicator dye 2′, 7′-dichlorofluorescin diacetate (DCF) (Fig. 7A). In addition, 10% of germlings from *basA1* cells possessed elevated ROS levels at 42 °C (Fig. 7C). These cells carry a point missense mutation in *basA*, the *A. nidulans SUR2* homologue of *S. cerevisiae*, which results in thermosensitivity of C4-hydroxylase, the enzyme catalyzing the conversion of dihydrosphingosine to phytosphingosine^29^. This increase in ROS levels was relieved by incubated *basA1* cells in the presence of the ROS scavenger N-acetyl cysteine (NAC) (Fig. 7B). Ιt is worth mentioning that in untreated wild type cells grown under these conditions, the percentage of ROS was extremely low. Consistent with the idea that sphingolipid depletion causes ROS accumulation, addition of phytosphingosine (PHS), a 4-hydroxylated LCB, only reduced ROS production in myriocin and not AbA treated cells. Notably, PHS administration in AbA-treated cells resulted in augmented ROS levels, suggesting that not only depletion of complex sphingolipids but also accumulation of LCBs can result to further production of ROS^30^ (Fig. 7B).

Sphingolipid biosynthesis in yeast is regulated by the Ypk1 kinase^18^,^19^,^21^,^31^,^32^,^33^. Interestingly, repression of YPK1 homologue in *A. nidulans*, using the regulatable promoter *niiA* (*niiA*::*ypkA* strain kindly provided by Dr. Goldman^34^) by ammonium resulted in a significant increase in ROS levels, evident after 12 h of growth in presence of ammonium as sole nitrogen source at 25 °C (Fig. 7D).

To further investigate the interrelationship of *ypkA* with sphingolipid biosynthesis and ROS production, double *niiA::ypkA basA1* mutant strains were constructed. These double mutants displayed a synthetic lethal phenotype when grown under repressing conditions at the non-permissive temperature (42 °C), although under inducing conditions at 42 °C *niiA::ypkA* partially suppressed *basA1* lethality (Fig. 7E). The above results confirm a genetic interaction between YpkA and the sphingolipid-mediated signaling pathway. In addition, ~35% of *niiA::ypkA basA1* double mutant germlings displayed elevated ROS under repressing conditions at 42 °C (Fig. 7F).

**Figure 8:**
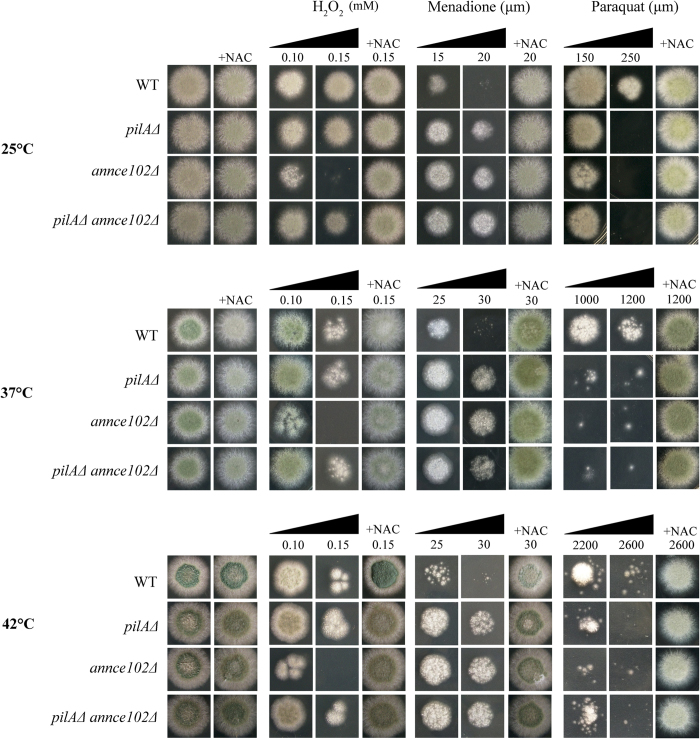
Growth of *pilA*Δ and/or *annce102*Δ mutants in the presence of oxidative agents. 5 μl of 10^7^/ml conidia of WT, *pilA*Δ*, annce102*Δ and *pilA*Δ *annce102*Δ cells spotted on MM and grown for 36–48 h in the presence of H_2_O_2_, paraquat and menadione, at concentrations as indicated, at 25, 37 or 42°, in the presence of urea as the sole nitrogen source.

Having established the conditions and tools, we sought to examine whether AnNce102 and PilA are directly involved in oxidative damage. In our analysis we also compared the use of two different selection markers to create the deletion of *annce102* in order to exclude possible marker effects (see Materials and Methods). In all cases, mutants lacking *annce102* showed similar growth, regardless of selection marker used. Cell viability of *A*. *nidulans* mutants growing in the presence of H_2_O_2_, menadione or paraquat was assayed (Fig. 8). Both drugs, menadione and paraquat are cycling agents that generate superoxide ions^35^. Interestingly, cells with deletion of *annce102* were more sensitive to H_2_O_2_ stress and *pilA*Δ and/or *annce102*Δ mutants were more resistant in the presence of menadione (Fig. 8). On the other hand, all mutant strains were found to be hypersensitive to paraquat (Fig. 8).

The above data show that ANnce102 or/and PilA play a role in protection against oxidative stress generated by paraquat and H_2_O_2_, also suggesting a role of the YpkA kinase in sphingolipid biosynthesis pathway and confirming the important role of YpkA and sphingolipids in redox homeostasis.

### AnNce102 and PilA genetically interact with YpkA and BasA, key players in sphingolipid metabolism

**Figure 9:**
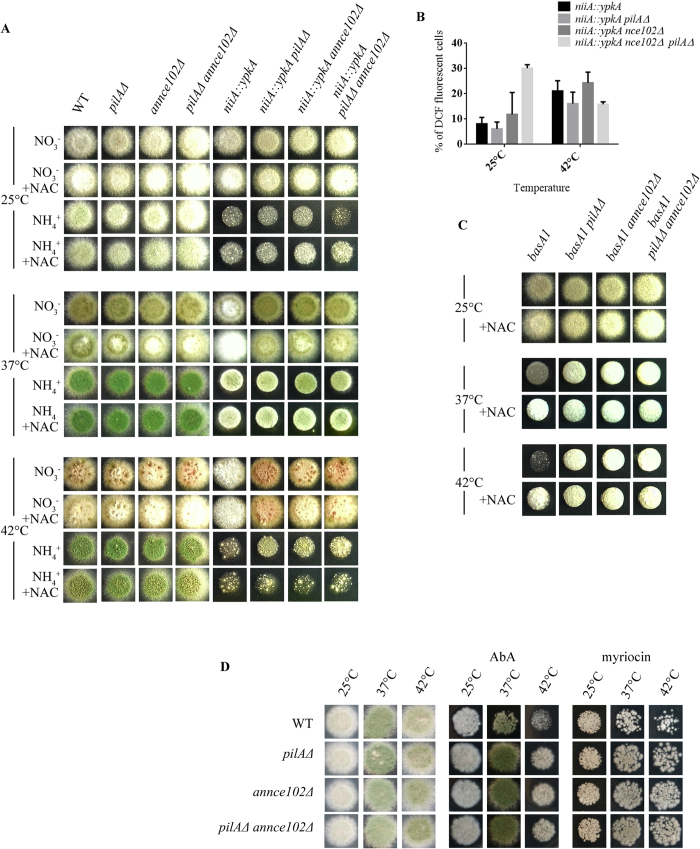
AnNce102 and PilA genetically interact with YpkA and sphingolipid biosynthesis pathway. (**A**) 5 μl of 10^5^ conidia of WT, *pilAΔ, annce102Δ, pilAΔ annce102Δ, niiA::ypkA*, *niiA::ypkA pilAΔ, niiA::ypkA annce102Δ* and *niiA::ypkA pilAΔ annce102Δ* cells spotted on MM and grown for 36–48 h at 25, 37 and 42 °C, under repressing or inducing conditions. (**B**) WT, *niiA::ypkA*, *niiA::ypkA pilAΔ, niiA::ypkA annce102Δ* and *niiA::ypkA pilAΔ annce102Δ* cells grown in MM for 12 h at 25 °C, under repressing or inducing conditions. Strains remain at 25 °C or shifted at 42 °C for additional 7 h, as indicated. ROS were determined and quantified as in Fig. 7A. (**C**) 5 μl of 10^5^ conidia of WT, *basA1, basA1 pilAΔ, basA1 annce102Δ, basA1 pilAΔ annce102Δ* cells spotted on MM and grown for 48 h at 25 or 42 °C, (D) 5 μl of 10^7^/ml conidia of WT, *pilA*Δ*, annce102*Δ and *pilA*Δ *annce102*Δ cells spotted on MM and grown for 36–48 h in the presence of 40 μg/ml myriocin or 1 μg/ml of AbA at 25, 37 and 42 °C.

In order to test whether deletion of PilA and AnNce102 affects ROS levels through regulation of YpkA activity, we created double and triple mutants of *pilAΔ* and/or *annce102*Δ with *niiA::ypkA.* Simultaneous deletion of both *pilA* and *annce102* results in impaired growth of a *niiA::ypkA* in ammonium at 25 °C, and, most importantly, seems to partially suppress the defects of *ypkA* downregulation at 42 °C (Fig. 9A). The growth of all mutants is well correlated with their intracellular ROS levels detected by DCF at 25 °C (Fig. 9B), where *niiA::ypkA annce102Δ pilAΔ* triple mutants display 3 fold more ROS at 25 °C. At 42 °C no statistically significant differences in ROS levels were detected. In order to test whether the above differences in growth and ROS accumulation are caused by the regulation of sphingolipid biosynthesis, we introduced the *pilA*Δ and/or *annce102Δ* mutations in a *basA1* strain. As shown in Fig. 9C, *basA1* mutants display slightly impaired growth at the semi-permissive temperature (37 °C), which is furthermore enhanced at non-permissive temperature (42 °C). Most remarkably, the double and triple mutants grow significantly better than the single *basA1*. Consistent with the above, *annce102*Δ and/or *pilA*Δ were found to be partially resistant to myriocin and AbA treatment at high temperatures (Fig. 9D). Curiously, addition of the ROS scavenger N-acetyl cysteine (NAC) effectively concealed any growth differences among *basA1, niiA::ypkA, annce102 and pilA* mutants observed above (Figs 9A,C), although no statistically significant differences in ROS levels were detected among mutants. Moreover, we quantitatively determine the inhibitory effects of the inhibitors of sphingolipid metabolism, myriocin and AbA, in WT, *pilAΔ, annce102*Δ, *pilAΔ annce102Δ* and *alcA::annce102* cells at 37 °C, in the presence of MM+2% glucose or MM+2% glycerol+ethanol. Myriocin was the least active against *A. nidulans* with a minimal inhibitory concentration (MIC) of 80 μg/ml in the presence of glucose and 60 μg/ml in the presence of glycerol+ethanol. The MIC of AbA was 4 μg/ml in the presence of glucose and 2 μg/ml in the presence of glycerol+ethanol. In these conditions we were not able to detect significant differences in the resistance or sensitivity of mutants compared to wild-type (Supplementary Fig. S4).

## Discussion

Several studies so far have documented the role of Nce102 proteins and despite their conservation among the ascomycetes^36^,^37^, the functional role of these proteins may diverge. For instance, in *Ashbya gossypii*, the orthologue of Nce102 despite colocalizing with eisosomes is not needed for eisosome stability and polar growth^38^. In *Aspergillus fumigatus* AfuNce102 deletion mutants showed a clear delay in conidiophore formation and severely affected sporulation, without affecting the virulence of the fungus^37^. Deletion of Nce102 from *Candida albicans* caused a 2-fold decrease in MCC/eisosomes. However, the cells displayed normal punctate localization of Lsp1-GFP and Sur7-GFP and showed decreased virulence by forming abnormal hyphae in mice^36^.

In this study, we present evidence that PilA and Nce102 are the main eisosomal organizers of *A. nidulans*. In particular, we showed that AnNce102 colocalizes with PilA at the PM and its temporal and spatial distribution resembles that of the SurG eisosomal protein. In addition, we observed that deletion of *pilA* has enormous impact on the stability of AnNce102 and SurG in germlings, where both proteins localized exclusively in vacuoles and endosomes. Despite the fact that increased endocytosis and enhanced direct vacuolar sorting of SurG and AnNce102 could both rationalize the data presented herein, we favor the hypothesis that the absence of PilA, which is mostly found at the plasma membrane, most probably destabilizes the otherwise stable foci of AnNce102 (and SurG) rather than it affects their intracellular trafficking. This is consistent with the proposed role of eisosomes in protecting Can1 from endocytosis in yeast^8^, which is however currently under debate^6^.

On the other hand, deletion of AnNce102 affects the older PilA/SurG eisosomal pool, resulting in fewer foci only in the head of germlings and without affecting eisosomes along hyphae. This phenotype resembles myriocin treatment and, similarly to *S. cerevisiae*^10^, can be overcome by overexpression of AnNce102. The above results suggest that two different populations of PilA foci exist in *A. nidulans* germlings; one at the head of the germlings that requires AnNce102 for proper organization and is sensitive to sphingolipid levels and another in the hyphae that does not respond to the above and possibly has different function and composition. Interestingly, deletion of *annce102* or *pilA* affects each other topology/foci number mostly in growing germlings. This temporal regulation could be related to the potential role(s) of eisosomes in sphingolipid and/or PI(4,5)P_2_ regulation^1–4 and present study^, recruitment of signaling molecules^5^ and the greater need of membrane biogenesis and compartmentalization in actively growing and not quiescent cells. Furthermore, overexpression of AnNce102 in *A. nidulans* led to ectopic localization of PilA foci into septa, a result that further confirms the crucial role of AnNce102 concerning eisosome organization, also suggesting a functional role of eisosomes related to septation. Consistent with this, deletion of *sur7* in *C. albicans* resulted in ectopic localization of the cdc12 septin from the bud neck^39^.

In *S. cerevisiae* Pil1 down-regulates the Ypk1 pathway^14^ and Nce102 has been implicated as a sensor for sphingolipid abundance and a negative regulator of pkh1 activity^10^. Our data support a subtle role for AnNce102 and PilA in the regulation of sphingolipid biosynthesis in *A. nidulans*. Indeed, we herein show that deletion of either *pilA* or *annce102* can partially suppress genetic (*basA1 and niiA*::*ypkA*) or pharmacological (myriocin and AbA) inhibition of sphingolipid biosynthesis at high temperatures (Fig. 9). Consistent with this, it has been shown previously that heat shock leads to a transient increase in sphingolipid production and that sphingolipid production is important for heat shock survival^40^,^41^. The fact that MIC measurements did not display significant differences, suggests a minor role of PilA and AnNce102 proteins in the regulation of sphingolipid metabolism (Fig. S4). An intriguing alternative explanation is that the difference observed in agar plates, in which *A. nidulans* colonies differentiate by asexual sporulation, cannot be observed by MIC measurements, which have been done in liquid media only allowing the growth of mycelia. Consistent with the above, the expression of *basA* was shown to increase upon begin of sexual and asexual development and the *basA1, barA and lagA* mutants have been shown to negatively affect the asexual/sexual ratio of sporulation in *A. nidulans*^29^, strongly indicating that asexual sporulation requires increased levels of sphingolipid biosynthesis. In light of the above, our results presented in Fig. 9 can be rationalized by developmental stage-specific genetic interaction of eisosomes and the sphingolipid biosynthesis pathway. In support of this, deletion of *ypkA* is known to result in very slow-growing, non-sporulating cells, while the defective growth of *a niiA::ypkA* strain in repressing conditions can be overcome by PHS supply^34^ suggesting that YpkA, possible through the regulation of sphingolipid metabolism, indeed participates in asexual sporulation.

Another important aspect related to Nce102 is its implication with oxidative stress^27^. Reactive oxygen species (ROS) are versatile molecular species and radicals that are poised at the core of a sophisticated network of signaling pathways and act as core regulators of cell physiology and cellular responses to environment^42^. ROS also help to monitor/modulate cellular processes that range from different cell fates^43^ to apoptosis^44^, from regulation of Apical Dominance^45^ to actin polarization^46^, suggesting that a kind of ‘ROS rheostat’ exists in cells. It is also known that oxidants can influence regulatory proteins to modulate sphingolipid metabolism and also appear to serve as upstream/downstream messengers for sphingolipid signaling^47^,^48^,^49^. In this study, we observed that, deletion of AnNce102 results in hypersensitivity to H_2_O_2_. However, treatment of *pilA*Δ and/or *annce102*Δ strains with 2 superoxide generators, had opposing effects on growth, where *pilA*Δ, *annce102*Δ and *pilA*Δ *annce102*Δ strains were more resistant in the presence of menadione and less resistant to paraquat. These results suggest a mechanism other than superoxide formation being responsible for this discrepancy. Moreover, we demonstrated that pharmacological (using myriocin and AbA) or genetic (using *basA1* mutant) inhibition of sphingolipid synthesis resulted in a significant increase in ROS levels. Interestingly, repression of YpkA (YPK1 homologue) expression resulted also in an increase in ROS, evident after 12 h of repression. The kinetics of this increases correlate well with the decreased radial growth, the delayed conidial germination, a deficiency in polar axis establishment and the intense branching observed after the germination of conidia of the *niiA::ypkA* strain grown under repressing conditions^34^. Furthermore, we showed that *niiA::ypkA basA1* double mutant strain displayed a synthetic lethal phenotype when grown under repressing conditions at the non-permissive temperature (42 °C), similar to synthetic lethality phenotype of *barA1 niiA::ypkA* mutants grown in the presence of ammonium at 37 °C^34^. In addition double mutants show higher ROS levels than single mutants at the same conditions, confirming a previous suggested role of YpkA kinase in sphingolipid biosynthesis pathway^34^ and suggesting an important role of YpkA and sphingolipids in redox homeostasis. Concerning the role of eisosomes/AnNce102 in YpkA/sphingolipid signaling we showed that *pilA*Δ and/or *annce102*Δ strains were partially resistant to pharmacological or genetic reduction of sphingolipid synthesis at high temperatures. Under these conditions, addition of the ROS scavenger N-acetyl cysteine (NAC) effectively concealed any growth differences among mutants, although no statistically significant differences in ROS levels were detected (Supplementary Fig. S5). A possible explanation could be that the ROS produced in these conditions cannot be detected by DCF, used in the present study. Interestingly we found *niiA::ypkA annce102Δ pilAΔ* triple mutant to be more sensitive at 25 °C under repressing conditions, and the only strain that displayed 3 fold more ROS than single or double mutants. Within this context, *nce102Δ* mutants of *C. albicans* were defective in forming hyphae and invading low concentrations of agar, likely due to a defect in actin organization, whereas Ypk1 has been proposed to regulate actin polarization by controlling ROS accumulation^46^. Since repression of YpkA mainly causes deficient polar axis establishment and intense branching^34^, an intriguing explanation could be that besides the role of PilA/AnNce102 in sphingolipid signaling, these proteins are likely to be involved in the regulation of cytoskeleton polarization through YpkA and a mechanism that involves ROS.

Previously we have shown that *pilA*Δ mutants, *pilA*Δ *pilB*Δ double mutants and *surG*Δ mutants displayed resistance to inhibitor of ergosterol synthesis, itraconazole^22^. In addition, it was recently shown that exogenous addition and/or improper accumulation of LCBs resulted in altered susceptibility to inhibitors of β -1,3-glucan synthesis^50^, echinocandins, in various fungi^51^. All these data support the possibility that eisosomal proteins modulate the mode of action of these groups of antifungal drugs by regulating sphingolipid biosynthesis rather than directly regulating β -1,3-glucan synthase as was hypothesized by Edlind and Katiyar^52^, making these proteins ideal candidates for studying sphingolipid signaling, antifungal resistance/susceptibility and their underlying mechanisms.

## Methods

The entire *annce102*, *mrvA*, and *mrvB* open reading frames (ORFs) (627 bp [AN7683], 570 bp [AN8278], and 747bp [AN8422], respectively) were replaced in appropriate strains (see Supplementary Table S1) by the *A. fumigatus, riboB (Afribo), pyrG* (*AfpyrG*) or *pyroA (Afpyro)* genes, using the fusion PCR gene replacement method^53^ and standard molecular cloning techniques. Recipient strains also carried a *nkuAΔ* mutation that results in a dramatically decreased frequency of heterologous integration events into the *A. nidulans* genome^54^. Cassettes containing the *annce102*::*sgfp* and *annce102*::*mrfp* fusions, expressed under the control of the endogenous promoter or under the *alcA* (alcohol dehydrogenenase gene) promoter, were constructed by joining three different PCR fragments, as previously described^53^. For the AnNce102-GFP and AnNce102-mRFP fusions, the GFP and mRFP were separated from the ORFs by a quintuple Gly-Ala di-peptide repeat (5GA linker). Cassettes for gene replacement were constructed and amplified using Kapa-HiFi (Kapa Biosystems) DNA polymerase and the primers of Supplementary Table S2. In-locus integration events in all transformants used in this study were confirmed by Southern analysis and PCR (see below).

Conventional PCR screen of the transformants was performed as previously described^55^, with the following modifications. Approximately 10^5^ conidiospores were suspended in 0.2 ml of extraction buffer (2% Triton X-100, 1% SDS, 10 0 mM NaCl, 1 mM EDTA, 10 mM Tris-HCl pH 8, 150–20 0 mg 0.01 mm glass beads), vortexed for 30 sec and incubated for 30 min at 60 °C, with vortexing every 10 min for 30 sec, prior to purification using 0.2 ml of phenol-SEVAG (phenol/chloroform/isoamyl alcohol, 25:24:1). After brief vortexing and centrifugation (12000 rpm, 5 min, 4 °C), the nucleic acids contained in 0.15 ml of supernatant were precipitated by adding equal volume of isopropanol, followed by centrifugation (12000 rpm, 15 min, RT), washed with 70% ethanol and resuspended in 50 ml RNase dH_2_O. 1 μl of each sample was used in 25 μl PCR reaction using KAPA Taq EXtra HotStart (Nippon Genetics Co., Ltd., Tokyo, Japan).

RNA samples were prepared as previously descripted^23^ RNA was isolated from quiescent conidia (0 h) and germlings (14 h), using the TRI Reagent (GIBCO-BRL) kit according to the instructions of the manufacturer. RNA samples were further purified according to the RNeasy Mini Protocol for RNA Cleanup, in the RNeasy Mini Kit (Qiagen). To avoid contamination with genomic DNA, 10 μg of each RNA sample were treated and cleaned up with TURBO DNA-freeTM kit (Ambion). The absence of DNA contamination was verified with conventional PCRs using specific-, surG, *annce102* and 18S rRNA set of primers and at least 3 μg of each RNA sample as template, which were amplified for 40 cycles. Primers were designed to cross at least an intron and expected to produce amplicons of 355 bp (*surG* cDNA), 478 bp (*annce102* cDNA), and 280 bp (*18S ribosomal RNA* gene). The quality of RNA was confirmed in a conventional 2% w/v agarose gel stained with ethidium bromide (Et-Br) (10 μg/ml). The concentration of each RNA sample was calculated using the nanodrop apparatus (ND-1000 Spectrophotometer) according to the instructions of the manufacturer. Approximately 3 μg of each RNA sample were used for reverse transcription using the SuperScriptTM II RNase H-reverse transcriptase (Invitrogen), according to the instructions of the manufacturer. For semi-quantitative analysis of transcript levels, cDNA was diluted 10-, 100-, 1000-fold. The number of cycles required to produce detectable difference in band intensity between dilutions (to avoid saturation) was determined with Et-Br staining.

*A. nidulans* cells were cultured in appropriately supplemented minimal medium (MM) at 25 °C, using 35 mm μ-Slides (Ibidi GmbH, Germany). Cells were imaged by the use of a Leica TCS SP5 (Leica Microsystems Ltd., Milton Keynes, UK) confocal microscope, with a Leica HCX PL APO CS 63x/1.4 NA oil immersion len. GFP and mRFP were excited with 488 and 531 nm laser lines, respectively. Mature endosomes/vacuoles were stained and visualized with CMAC (7-amino-4-chloromethyl-coumarin) (Molecular Probes) as previously described (Gournas *et al.*, 2010). Septa were stained with calcofluor white (CFW) (Sigma-Aldrich, St. Louis, MO) as descripted by (McIntyre *et al.*, 2001).

For most figures, representative images of the equatorial sections and/or volumetric views of confocal stacks are shown. Images were acquired sequentially as Z-series (step size 0.2 μm) and processed/analyzed using the Fiji/imagej software (http://imagej.nih.gov/ij/download.html) or the Imaris 7.2.4 (Bitplane AG, Zurich, Switzerland). For colocalization assessment *z*-stacks were deconvolved using a three-dimensional (3D) blind algorithm [Autoquant X (Media Cybernetics, Silver Spring, MD)]. Colocalization was measured using Imaris software package (Bitplane, Zurich, Switzerland). The threshold of each channel used to quantify colocalization was determined automatically, according to the method of Costes^56^. Colocalization was defined as the overlap of two channels in three dimensions and was calculated by the program automatically. Colocalization was analyzed by using Manders colocalization coefficients (M1 and M2) that describe the colocalization of molecules with respect to an individual channel, and increase from 0 to 1 with rising colocalization and visual inspection of the spatial relations (intensity spatial profile). 2D histograms (plotted pixels of a two-channel image) were obtained using Imaris (Bitplane). Colocalization was represented as new channel (colocalization channel) corresponding to the colocalized voxels. PilA foci in germlings were counted using spot detection tool of the Imaris (Bitlane) software. A minimum diameter of 0.3 μm and an automatic threshold detection of the software were used, adjusted manually to identify all visible PilA foci.

Time-lapse experiments were conducted at 26 °C. Cells were imaged by the use of Leica TCS SP5 (Leica Microsystems Ltd., Milton Keynes, UK) confocal microscope with a Leica HCX PL APO CS 63x/1.4 NA oil immersion lens. The images were analyzed with the Imaris software package (Bitplane, Zurich, Switzerland).

Total protein extracts were prepared as previously described^23^,^57^, with the following modifications. Approximately 500 mg of liquid N-grinded conidiospores or young mycelia (14 h, 25 °C) were resuspended in 1.5 ml ice-cold precipitation buffer (50 mM Tris-HCl pH 7, 50 mM NaCl, 1 mM EDTA), supplemented with a protease inhibitor cocktail (Sigma) and 1 mM phenylmethylsulfonyl fluoride (PMSF). TCA was added (1/8 of total V), the suspension was briefly vortexed, followed by centrifugation (15000 g, 10 min, 4 °C). The pellet was washed twice with ice cold acetone, heat-dried and dissolved in 0.5 ml extraction buffer (150 mM Tris-HCl pH 8, 50 mM NaCl, 1% w/v SDS, 1mM EDTA and protease inhibitor cocktail). Protein concentrations were determined by the method of Bradford. In each case 30–50 μg protein were fractionated on a 10% (w/v) SDS-polyacrylamide gel and electroblotted (Mini Protean Tetra cell, Bio-Rad) onto a polyvinylidene difluoride (PVDF) membrane (Macherey-Nagel, Lab Supplies Scientific SA, Hellas) for immunodetection. The membrane was treated with 2% (w/v) non-fat dry milk, and immunodetection was performed using a primary mouse anti-GFP monoclonal antibody (Roche), a mouse anti-actin monoclonal (C4) antibody (MP Biomedicals Europe, Lab Supplies Scientific SA, Hellas) and a secondary rabbit anti-mouse IgG horseradish peroxidase (HRP)-linked antibody (Cell Signaling). Blots were developed by the chemiluminescent method using the LumiSensor Chemiluminescent HRP Substrate kit (GenScript USA Inc, Lab Supplies Scientific SA), an enhanced chemiluminescence reagent (Amersham Bioscience) and SuperRX Fuji medical X-Ray films (FujiFILM Europe, Lab Supplies Scientific SA, Hellas).

Myriocin and Aureobasidin A susceptibility assays. The susceptibility assays were performed by measuring the MIC of each molecule in 96-well flat bottom plates, using a slightly modified protocol previously described^58^. Briefly, the assay mixture was prepared by adding 1 volume of conidial suspension (10^5^ conidia ml^−1^ in 0.05% Tween 20) to 12 volumes of assay medium (MM+2% glucose or MM+2% glycerol+ethanol). Two-fold dilutions of each drug were prepared with this assay mixture. The plates were incubated at 37 °C for 24 h. After incubation, the culture medium was removed from each well and the biofilms were washed three times with Milli-Q water. 130 ml of 0.01% (w/v) crystal violet solution (Sigma Aldrich) was added to each well for 20 min at room temperature. The solution was then removed and the biofilms were washed until the supernatant was clear. The plates were air-dried and the absorbance at 560 nm was measured in a Tecan Infinite M-1000 PRO plate reader (Tecan Systems Inc., San Jose, CA).

## Additional Information

**How to cite this article**: Athanasopoulos, A. *et al.* Characterization of AnNce102 and its role in eisosome stability and sphingolipid biosynthesis. *Sci. Rep.* **5**, 15200; doi: 10.1038/srep15200 (2015).

## Supplementary Material

Supplementary Information

## References

[b1] Bagnat M. & Simons K. Lipid rafts in protein sorting and cell polarity in budding yeast Saccharomyces cerevisiae. Biol. Chem. 383, 1475–1480 (2002). 12452424 10.1515/BC.2002.169

[b2] Lingwood D. & Simons K. Lipid Rafts As a Membrane-Organizing Principle. Sci. January 1 2010 327, 46–50 (2010). 10.1126/science.1174621 20044567

[b3] Spira F. *et al.* Patchwork organization of the yeast plasma membrane into numerous coexisting domains. Nat. Cell Biol. 14, 640–648 (2012). 22544065 10.1038/ncb2487

[b4] Douglas L. M. & Konopka J. B. Fungal Membrane Organization: The Eisosome Concept. Annu. Rev. Microbiol. 68, 140626173329002 (2014). 10.1146/annurev-micro-091313-103507 25002088

[b5] Malínská K., Malínskỳ J., Opekarová M. & Tanner W. Visualization of protein compartmentation within the plasma membrane of living yeast cells. Mol. Biol. Cell 14, 4427–4436 (2003). 14551254 10.1091/mbc.E03-04-0221 PMC266762

[b6] Brach T., Specht T. & Kaksonen M. Reassessment of the role of plasma membrane domains in the regulation of vesicular traffic in yeast. J. Cell Sci. 124, 328–337 (2011). 21224391 10.1242/jcs.078519

[b7] Grossmann G., Opekarová M., Malinsky J., Weig-Meckl I. & Tanner W. Membrane potential governs lateral segregation of plasma membrane proteins and lipids in yeast. EMBO J. 26, 1–8 (2006). 17170709 10.1038/sj.emboj.7601466 PMC1782361

[b8] Grossmann G. *et al.* Plasma membrane microdomains regulate turnover of transport proteins in yeast. J. Cell Biol. 183, 1075–1088 (2008). 19064668 10.1083/jcb.200806035 PMC2600745

[b9] Young M. E. *et al.* The Sur7p family defines novel cortical domains in Saccharomyces cerevisiae, affects sphingolipid metabolism, and is involved in sporulation. Mol. Cell. Biol. 22, 927–934 (2002). 11784867 10.1128/MCB.22.3.927-934.2002 PMC133540

[b10] Frohlich F. *et al.* A genome-wide screen for genes affecting eisosomes reveals Nce102 function in sphingolipid signaling. J. Cell Biol. 185, 1227–1242 (2009). 19564405 10.1083/jcb.200811081 PMC2712959

[b11] Ziółkowska N. E., Christiano R. & Walther T. C. Organized living: formation mechanisms and functions of plasma membrane domains in yeast. Trends Cell Biol. 22, 151–158 (2012). 22245053 10.1016/j.tcb.2011.12.002

[b12] Luo G., Gruhler A., Liu Y., Jensen O. N. & Dickson R. C. The Sphingolipid Long-chain Base-Pkh1/2-Ypk1/2 Signaling Pathway Regulates Eisosome Assembly and Turnover. J. Biol. Chem. 283, 10433–10444 (2008). 18296441 10.1074/jbc.M709972200 PMC2447625

[b13] Walther T. C. *et al.* Eisosomes mark static sites of endocytosis. Nature 439, 998–1003 (2006). 16496001 10.1038/nature04472

[b14] Zhang X. Pil1p and Lsp1p Negatively Regulate the 3-Phosphoinositide-dependent Protein Kinase-like Kinase Pkh1p and Downstream Signaling Pathways Pkc1p and Ypk1p. J. Biol. Chem. 279, 22030–22038 (2004). 15016821 10.1074/jbc.M400299200

[b15] Hannun Y. A. & Obeid L. M. Principles of bioactive lipid signalling: lessons from sphingolipids. Nat. Rev. Mol. Cell Biol. 9, 139–150 (2008). 18216770 10.1038/nrm2329

[b16] Kajiwara K. *et al.* Perturbation of sphingolipid metabolism induces endoplasmic reticulum stress-mediated mitochondrial apoptosis in budding yeast: Sphingolipids regulate ER stress-mediated yeast apoptosis. Mol. Microbiol. 86, 1246–1261 (2012). 23062268 10.1111/mmi.12056

[b17] Berchtold D. *et al.* Plasma membrane stress induces relocalization of Slm proteins and activation of TORC2 to promote sphingolipid synthesis. Nat. Cell Biol. 14, 542–547 (2012). 22504275 10.1038/ncb2480

[b18] Niles B. J. & Powers T. Plasma membrane proteins Slm1 and Slm2 mediate activation of the AGC kinase Ypk1 by TORC2 and sphingolipids in S. cerevisiae. Cell Cycle 11, 3745–3749 (2012). 22895050 10.4161/cc.21752 PMC3495817

[b19] Roelants F. M., Breslow D. K., Muir A., Weissman J. S. & Thorner J. Protein kinase Ypk1 phosphorylates regulatory proteins Orm1 and Orm2 to control sphingolipid homeostasis in Saccharomyces cerevisiae. Proc. Natl. Acad. Sci. 108, 19222–19227 (2011). 22080611 10.1073/pnas.1116948108 PMC3228448

[b20] Sun Y. *et al.* Orm protein phosphoregulation mediates transient sphingolipid biosynthesis response to heat stress via the Pkh-Ypk and Cdc55-PP2A pathways. Mol. Biol. Cell 23, 2388–2398 (2012). 22535525 10.1091/mbc.E12-03-0209 PMC3374756

[b21] Muir A., Ramachandran S., Roelants F. M., Timmons G. & Thorner J. TORC2-dependent protein kinase Ypk1 phosphorylates ceramide synthase to stimulate synthesis of complex sphingolipids. eLife 3, e03779 (2014). 10.7554/eLife.03779 PMC4217029 25279700

[b22] Vangelatos I. *et al.* Eisosome Organization in the Filamentous AscomyceteAspergillus nidulans. Eukaryot. Cell 9, 1441–1454 (2010). 20693301 10.1128/EC.00087-10 PMC2950425

[b23] Athanasopoulos A., Boleti H., Scazzocchio C. & Sophianopoulou V. Eisosome distribution and localization in the meiotic progeny of Aspergillus nidulans. Fungal Genet. Biol. 53, 84–96 (2013). 23395641 10.1016/j.fgb.2013.01.002

[b24] Erpapazoglou Z., Kafasla P. & Sophianopoulou V. The product of the SHR3 orthologue of Aspergillus nidulans has restricted range of amino acid transporter targets. Fungal Genet. Biol. 43, 222–233 (2006). 16531082 10.1016/j.fgb.2005.11.006

[b25] Heidler S. A. & Radding J. A. The AUR1 gene in Saccharomyces cerevisiae encodes dominant resistance to the antifungal agent aureobasidin A (LY295337). Antimicrob. Agents Chemother. 39, 2765–2769 (1995). 8593016 10.1128/aac.39.12.2765 PMC163026

[b26] Felenbok B., Flipphi M. & Nikolaev I. Ethanol catabolism in Aspergillus nidulans: A model system for studying gene regulation. Prog. Nucleic Acid Res. Mol. Biol. 69, 149–204 (2001). 11550794 10.1016/s0079-6603(01)69047-0

[b27] Desmyter L. *et al.* Nonclassical export pathway: overexpression of NCE102 reduces protein and DNA damage and prolongs lifespan in an SGS1 deficient Saccharomyces cerevisiae. Biogerontology 8, 527–535 (2007). 17415679 10.1007/s10522-007-9095-5

[b28] Cheng J., Park T.-S., Fischl A. S. & Ye X. S. Cell Cycle Progression and Cell Polarity Require Sphingolipid Biosynthesis in Aspergillus nidulans. Mol. Cell. Biol. 21, 6198–6209 (2001). 11509663 10.1128/MCB.21.18.6198-6209.2001 PMC87337

[b29] Li S., Bao D., Yuen G., Harris S. D. & Calvo A. M. basA Regulates Cell Wall Organization and Asexual/Sexual Sporulation Ratio in Aspergillus nidulans. Genetics 176, 243–253 (2007). 17409079 10.1534/genetics.106.068239 PMC1893078

[b30] Cheng J., Park T. S., Chio L. C., Fischl A. S. & Xiang S. Y. Induction of apoptosis by sphingoid long-chain bases in Aspergillus nidulans. Mol. Cell. Biol. 23, 163–177 (2003). 12482970 10.1128/MCB.23.1.163-177.2003 PMC140675

[b31] Liu K. The Sphingoid Long Chain Base Phytosphingosine Activates AGC-type Protein Kinases in Saccharomyces cerevisiae Including Ypk1, Ypk2, and Sch9. J. Biol. Chem. 280, 22679–22687 (2005). 15840588 10.1074/jbc.M502972200

[b32] Niles B. J., Mogri H., Hill A., Vlahakis A. & Powers T. Plasma membrane recruitment and activation of the AGC kinase Ypk1 is mediated by target of rapamycin complex 2 (TORC2) and its effector proteins Slm1 and Slm2. Proc. Natl. Acad. Sci. 109, 1536–1541 (2012). 22307609 10.1073/pnas.1117563109 PMC3277121

[b33] Sun Y. *et al.* Sli2 (Ypk1), a homologue of mammalian protein kinase SGK, is a downstream kinase in the sphingolipid-mediated signaling pathway of yeast. Mol. Cell. Biol. 20, 4411–4419 (2000). 10825204 10.1128/mcb.20.12.4411-4419.2000 PMC85808

[b34] Colabardini A. C., Brown N. A., Savoldi M., Goldman M. H. S. & Goldman G. H. Functional Characterization of Aspergillus nidulans ypkA, a Homologue of the Mammalian Kinase SGK. PLoS ONE 8, e57630 (2013). 23472095 10.1371/journal.pone.0057630 PMC3589345

[b35] Lamarre C., LeMay J.-D., Deslauriers N. & Bourbonnais Y. Candida albicans Expresses an Unusual Cytoplasmic Manganese-containing Superoxide Dismutase (SOD3 Gene Product) upon the Entry and during the Stationary Phase. J. Biol. Chem. 276, 43784–43791 (2001). 11562375 10.1074/jbc.M108095200

[b36] Douglas L. M., Wang H. X. & Konopka J. B. The MARVEL Domain Protein Nce102 Regulates Actin Organization and Invasive Growth of Candida albicans. mBio 4, e00723–13–e00723–13 (2013). 24281718 10.1128/mBio.00723-13 PMC3870249

[b37] Khalaj V., Azizi M., Enayati S., Khorasanizadeh D. & Ardakani E. M. NCE102 homologue in Aspergillus fumigatus is required for normal sporulation, not hyphal growth or pathogenesis. FEMS Microbiol. Lett. 329, 138–145 (2012). 22289033 10.1111/j.1574-6968.2012.02513.x

[b38] Seger S., Rischatsch R. & Philippsen P. Formation and stability of eisosomes in the filamentous fungus Ashbya gossypii. J. Cell Sci. 124, 1629–1634 (2011). 21525038 10.1242/jcs.082487

[b39] Alvarez F. J., Douglas L. M., Rosebrock A. & Konopka J. B. The Sur7 protein regulates plasma membrane organization and prevents intracellular cell wall growth in Candida albicans. Mol. Biol. Cell 19, 5214–5225 (2008). 18799621 10.1091/mbc.E08-05-0479 PMC2592640

[b40] Cowart L. A. Roles for Sphingolipid Biosynthesis in Mediation of Specific Programs of the Heat Stress Response Determined through Gene Expression Profiling. J. Biol. Chem. 278, 30328–30338 (2003). 12740364 10.1074/jbc.M300656200

[b41] Jenkins G. M. *et al.* Involvement of yeast sphingolipids in the heat stress response of Saccharomyces cerevisiae. J. Biol. Chem. 272, 32566–32572 (1997). 9405471 10.1074/jbc.272.51.32566

[b42] Bhattacharjee S. The Language of Reactive Oxygen Species Signaling in Plants. J. Bot. 2012, e985298 (2012).

[b43] Maryanovich M. & Gross A. A ROS rheostat for cell fate regulation. Trends Cell Biol. 23, 129–134 (2013). 23117019 10.1016/j.tcb.2012.09.007

[b44] Hideg E. *et al.* Detection of singlet oxygen and superoxide with fluorescent sensors in leaves under stress by photoinhibition or UV radiation. Plant Cell Physiol. 43, 1154–1164 (2002). 12407195 10.1093/pcp/pcf145

[b45] Semighini C. P. & Harris S. D. Regulation of Apical Dominance in Aspergillus nidulans Hyphae by Reactive Oxygen Species. Genetics 179, 1919–1932 (2008). 18689883 10.1534/genetics.108.089318 PMC2516069

[b46] Niles B. J. & Powers T. TOR complex 2–Ypk1 signaling regulates actin polarization via reactive oxygen species. Mol. Biol. Cell 25, 3962–3972 (2014). 25253719 10.1091/mbc.E14-06-1122 PMC4244204

[b47] Castillo S. S., Levy M., Thaikoottathil J. V. & Goldkorn T. Reactive nitrogen and oxygen species activate different sphingomyelinases to induce apoptosis in airway epithelial cells. Exp. Cell Res. 313, 2680–2686 (2007). 17498692 10.1016/j.yexcr.2007.04.002

[b48] Nikolova-Karakashian M. N. & Reid M. B. Sphingolipid Metabolism, Oxidant Signaling, and Contractile Function of Skeletal Muscle. Antioxid. Redox Signal. 15, 2501–2517 (2011). 21453197 10.1089/ars.2011.3940 PMC3176343

[b49] Niles B. J., Joslin A. C., Fresques T. & Powers T. TOR Complex 2-Ypk1 Signaling Maintains Sphingolipid Homeostasis by Sensing and Regulating ROS Accumulation. Cell Rep. (2014). 10.1016/j.celrep.2013.12.040 PMC3985744 24462291

[b50] Douglas C. M. Fungal β(1,3)-d-glucan synthesis. Med. Mycol. 39, 55–66 (2001). 11800269 10.1080/mmy.39.1.55.66

[b51] Healey K. R., Challa K. K., Edlind T. D. & Katiyar S. K. Sphingolipids mediate differential echinocandin susceptibility in Candida albicans and Aspergillus nidulans. *Antimicrob*. Agents Chemother. AAC. 04667–14 (2015). 10.1128/AAC.04667-14 PMC4432167 25824222

[b52] Edlind T. D. & Katiyar S. K. The echinocandin ‘target’ identified by cross-linking is a homolog of Pil1 and Lsp1, sphingolipid-dependent regulators of cell wall integrity signaling. Antimicrob. Agents Chemother. 48, 4491 (2004). 15504893 10.1128/AAC.48.11.4491.2004 PMC525446

[b53] Szewczyk E. *et al.* Fusion PCR and gene targeting in Aspergillus nidulans. Nat. Protoc. 1, 3111–3120 (2007). 17406574 10.1038/nprot.2006.405

[b54] Nayak T. A Versatile and Efficient Gene-Targeting System for Aspergillus nidulans. Genetics 172, 1557–1566 (2005). 16387870 10.1534/genetics.105.052563 PMC1456264

[b55] Hervas-Aguilar A., Rodriguez J. M., Tilburn J., Arst H. N. & Penalva M. A. Evidence for the Direct Involvement of the Proteasome in the Proteolytic Processing of the Aspergillus nidulans Zinc Finger Transcription Factor PacC. J. Biol. Chem. 282, 34735–34747 (2007). 17911112 10.1074/jbc.M706723200

[b56] Costes S. V. *et al.* Automatic and Quantitative Measurement of Protein-Protein Colocalization in Live Cells. Biophys. J. 86, 3993–4003 (2004). 15189895 10.1529/biophysj.103.038422 PMC1304300

[b57] Gournas C., Evangelidis T., Athanasopoulos A., Mikros E. & Sophianopoulou V. The Aspergillus nidulans proline permease as a model for understanding the factors determining substrate binding and specificity of fungal amino acid transporters. J. Biol. Chem. jbc. M114.612069 (2015). 10.1074/jbc.M114.612069 PMC4358254 25572393

[b58] Briard B. *et al.* Pseudomonas aeruginosa manipulates redox and iron homeostasis of its microbiota partner Aspergillus fumigatus via phenazines. Sci. Rep. (2015) Feb 10;5:8220. 10.1038/srep08220. PMC5389140 25665925

[b59] Amillis S. *et al.* Transcription of purine transporter genes is activated during the isotropic growth phase of Aspergillus nidulans conidia. Mol. Microbiol. 52, 205–216 (2004). 15049821 10.1046/j.1365-2958.2003.03956.x

