# Supplementary material for: Characterization of AnNce102 and its role in eisosome stability and sphingolipid biosynthesis
Source: Sci Rep. 2015 Oct 15;5:15200. doi: 10.1038/srep15200 (PMC4606592; doi:10.1038/srep15200)
Supplement: Supplementary Information [file srep15200-s1.pdf]

Supplementary Information

Characterization of AnNce102 and its role in eisosome stability and sphingolipid biosynthesis

Alexandros Athanasopoulos[1], Christos Gournas[1#], Sotiris Amillis[2], Vicky Sophianopoulou[1*]

[1]Institute of Biosciences and Applications, Microbial Molecular Genetics Laboratory, National Center for Scientific Research, Demokritos (NCSRD), Athens, Greece

[2]Faculty of Biology, University of Athens, Panepistimioupolis 15781, Athens, Greece

# Present address: Molecular Physiology of the Cell, Université Libre de Bruxelles, IBMM, 6041 Gosselies, Belgium

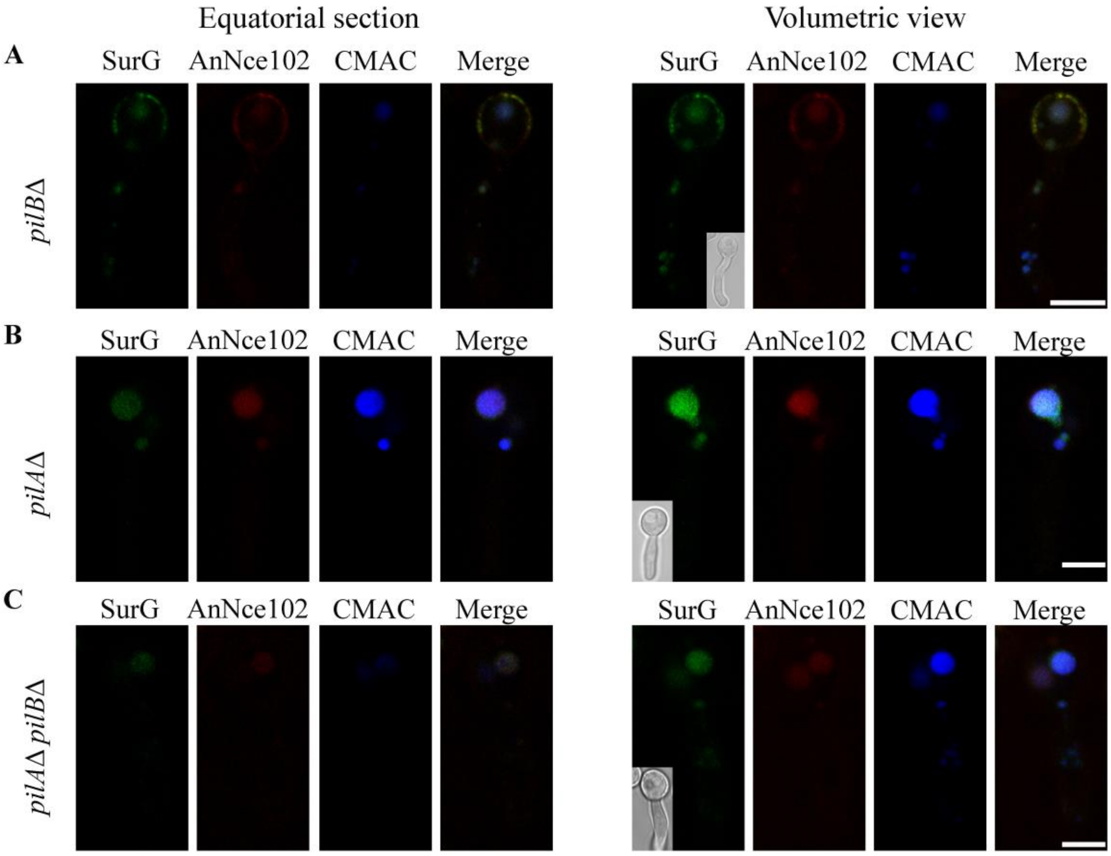

Supplementary Figure S1 | Deletion of *pilB* does not affect the membrane localization of Nce102 and SurG in germlings. Confocal images of strains expressing AnNce102-mRFP and SurG-GFP in (A) *pilB*Δ, (B) *pilA*Δ and (C) *pilB*Δ *pilA*Δ mutants. The insets show phase contrast images of optical sections. Images represent equatorial sections and volumetric views of confocal stacks generated by Imaris. Scale Bars 5 µm.

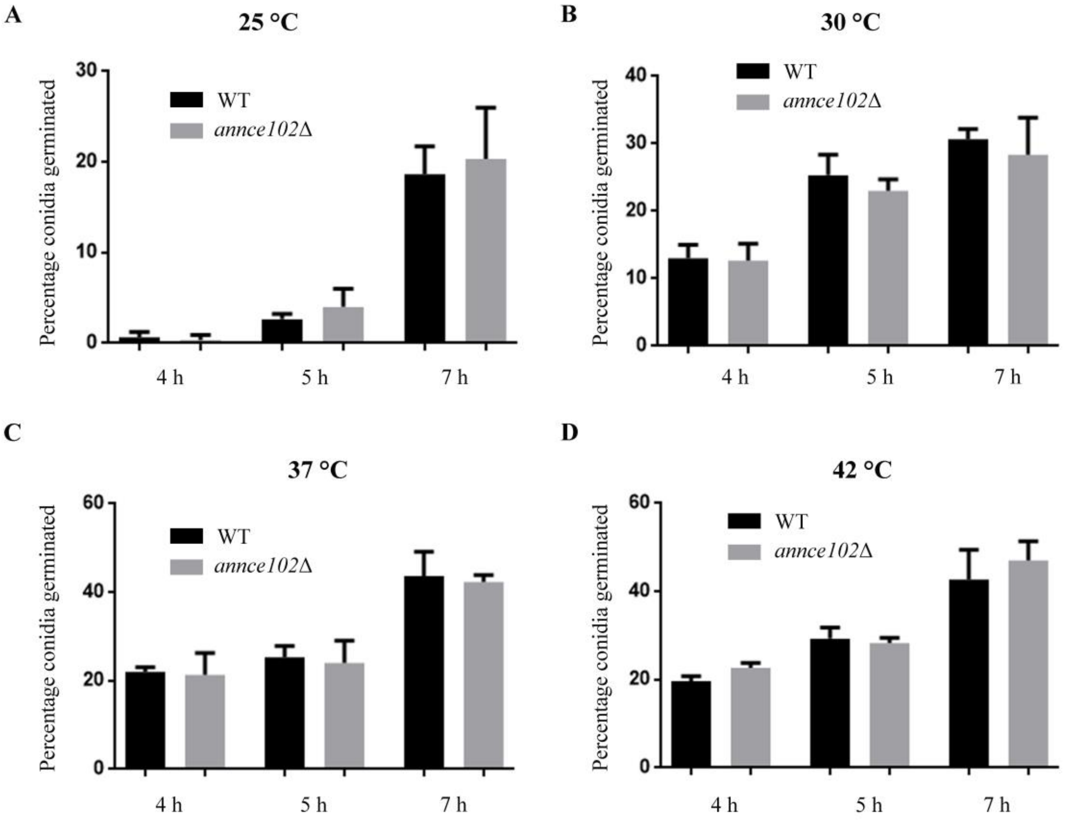

Supplementary Figure S2 | Percentage of germination wild type and *annce102*Δ conidia, at different temperatures. Conidial suspensions of wild type and *annce102*Δ cells were inoculated onto glass coverslips in the presence of 5 mM urea and 1% (w/v) glucose as the sole nitrogen and carbon sources, respectively and incubated at 25, 30, 37 and 42 °C (**A**, **B**, **C** and **D**, respectively). Samples were collected at several time points (4, 5 and 7 h) and were observed using a 40× 1.20 NA objective lens on an Axioplan 2 microscope (Carl Zeiss, Inc.). Images were acquired with a ZeissMRC5 digital camera using the AxioVs40 V4.40.0 software. Images were then processed with ImageJ (NIH) and annotated with the Photoshop CS6 software (Adobe). Quantification represents percentage of 200 – 300 cells, from at least three independent experiments, with averages presented as mean ± SEM.

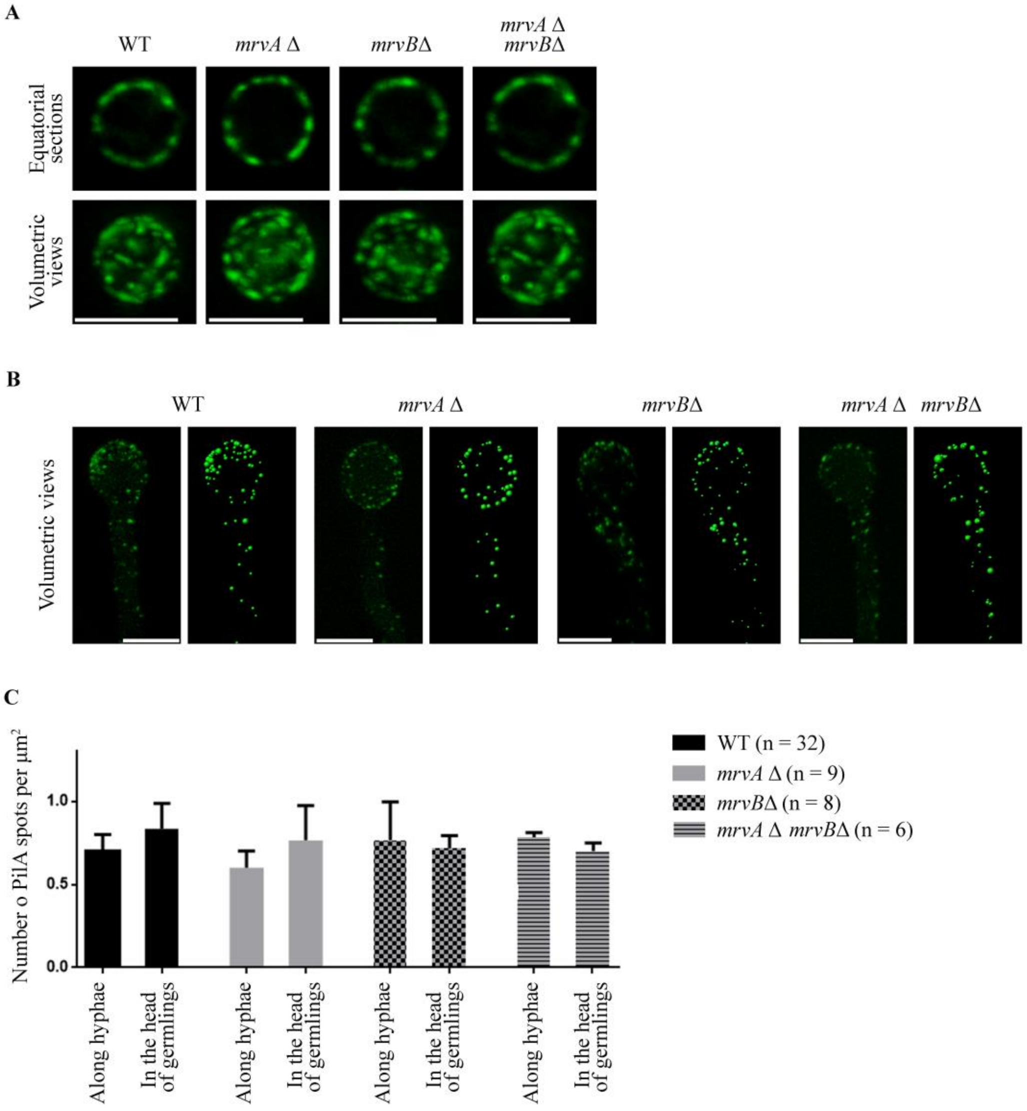

Supplementary Figure S3 | Deconvoluted confocal images of PilA in (**A**) quiescent conidia (0 h) and (**B**) germlings (14 h), of WT, *mrvA*Δ, *mrvB*Δ and *mrvA*Δ *mrvB*Δ cells. The number of PilA foci in germlings were determined and quantified as in (Fig. 4) and shown in (**C**) as a histogram. n represents the number of cells examined from at least two independent experiments, with averages presented as mean ± SEM. Scale Bars 5 μm.

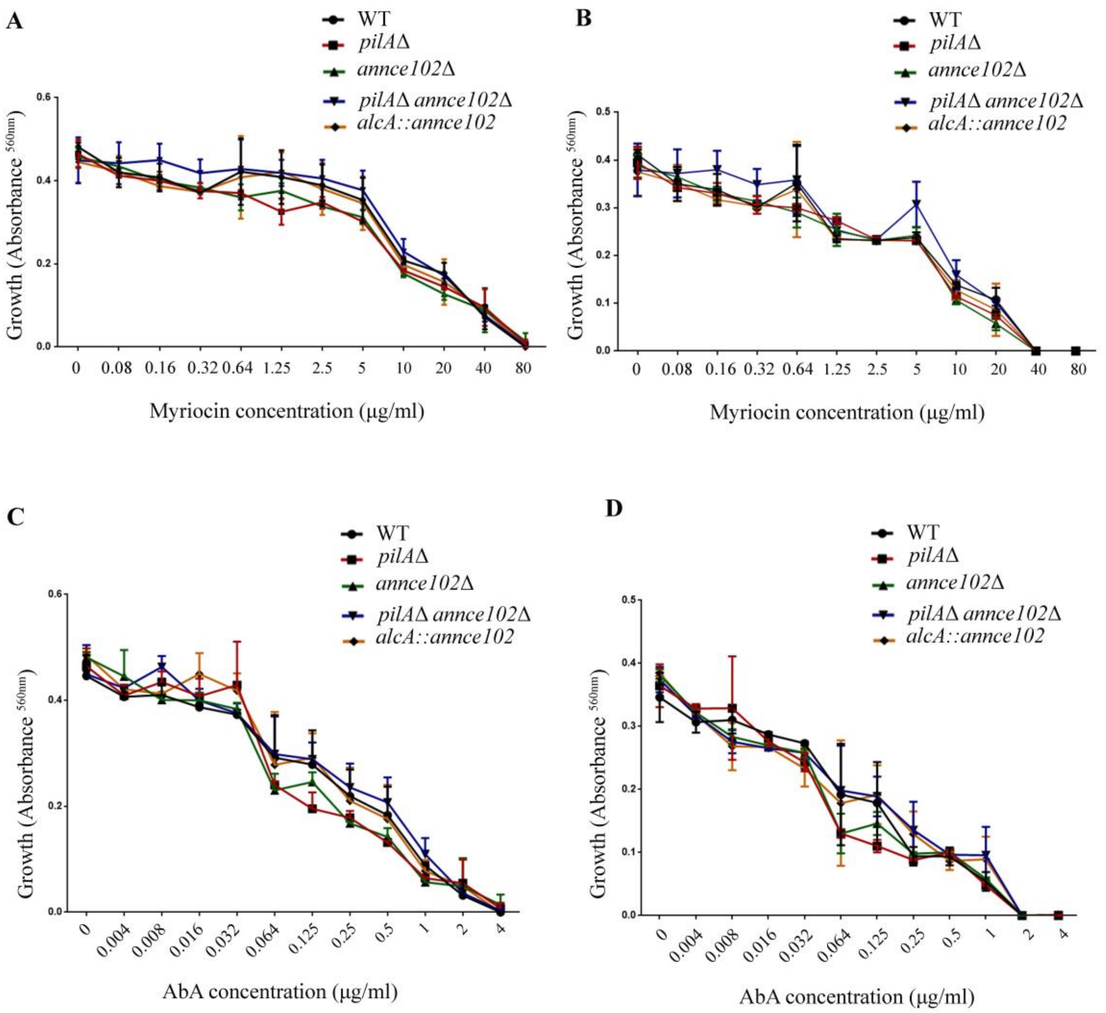

Supplementary Figure S4 | MIC of myriocin and Aureobasidin A against wild type and *pilAΔ*, *annce102*Δ, *pilAΔ annce102Δ* and *alcA::annce102* mutant cells of *A. nidulans*. Growth of cells was determined in the presence of increasing concentrations of the two inhibitors of sphingolipid metabolism. Mycelial growth was estimated by reading the absorbance at 560 nm, using the crystal violet method, after 24 h in the presence of (**A** and **C**) MM+2% glucose (repressing conditions of *alcA::annce102*) or (**B** and **D**) MM+2% glycerol+EtOH (inducing conditions of *alcA::annce102*) at 37 °C. Data are presented as mean ± SEM obtained from two independent experiments.

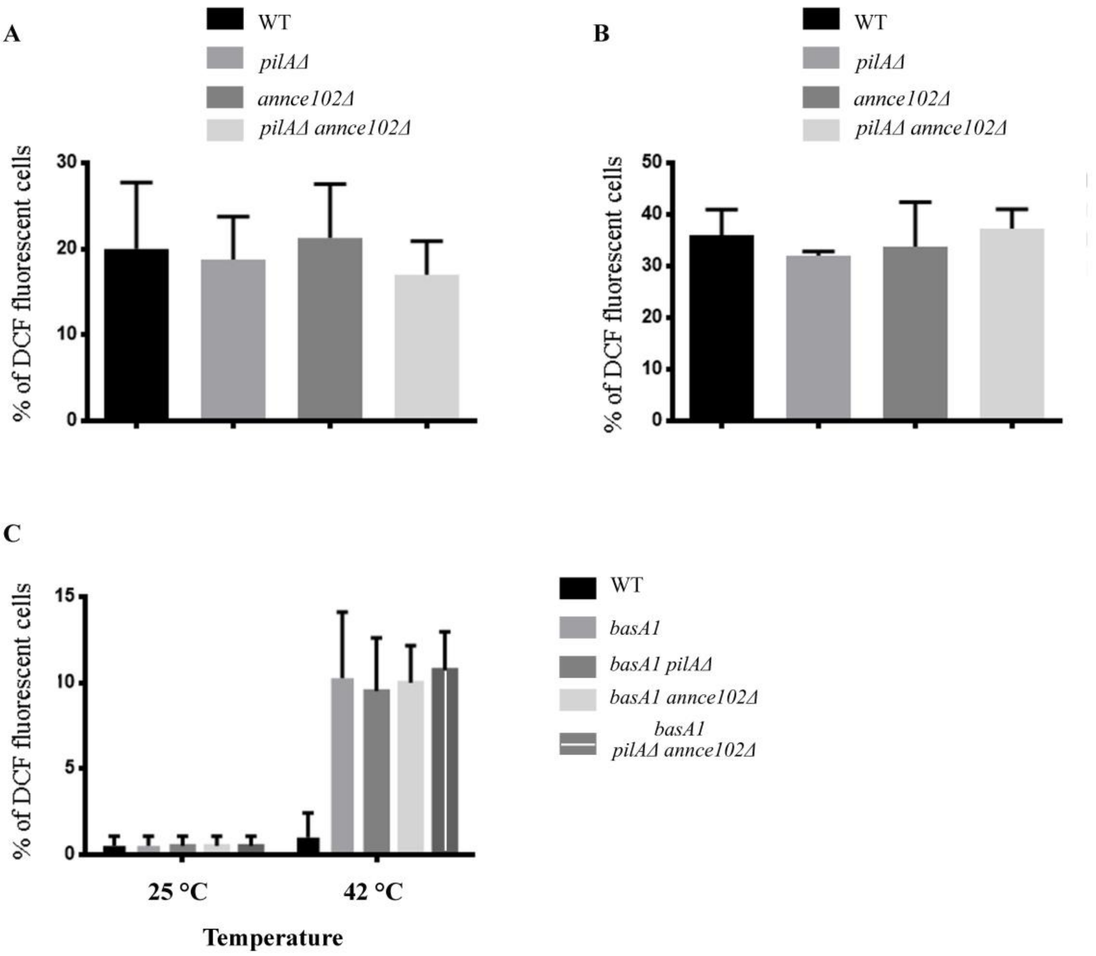

Supplementary Figure S5 | **ROS accumulation by sphingolipid depletion.** WT, *pilAΔ*, *annce102Δ* and *pilAΔ annce102Δ* cells grown in MM for 12 h and then treated for 7 h with (**A**) 40 μg/ml myriocin and (**B**) 2 μg/ml AbA. (**C**) WT, *basA1, basA1 pilAΔ*, *basA1 annce102Δ*, *basA1 pilAΔ annce102Δ* cells grown in MM for 12 h at 25 °C. Strains were remained at 25 °C or shifted at 42 °C for additional 7 h of growth. ROS were determined and quantified as in Fig. 7A.

Supplementary Table S1 | *Aspergillus nidulans* strains used and constructed in this study

**Strain**	**Genotype**	**Source**
wt	*pabaA1*	Fungal Genetics Stock Center
wt	*pyroA4*	This work
niiA::ypkA	*pyroA4; chaA1; argB2; Δnku::argB; niiA::ypkA::pyrG*	Colabardini et al., 2013
8-145	*pabaA1; biA; basA1*	Li et al., 2006
nkuAΔ pyrG89 riboB2	*pyrG89; argB2; nkuAΔ::argB$^+$; pabaB22; riboB2*	Vangelatos et al., 2010
nkuAΔ pyrG89 pyroA4	*pyrG89; argB2; nkuAΔ::argB$^+$; pyroA4*	Vangelatos et al., 2010
VS79	*pyrG89; argB2; nkuAΔ::argB$^+$; pabaB22; pilA::sgfp::AfpyrG$^+$; riboB2*	Vangelatos et al., 2010
VS81	*pyrG89; surG::sgfp::AfpyrG$^+$; argB2; nkuAΔ::argB$^+$; pabaB22; riboB2*	Vangelatos et al., 2010
VS83	*pyrG89; argB2; nkuAΔ::argB$^+$; pyroA4; pilA::mrfp::AfpyrG$^+$*	Vangelatos et al., 2010
VS91	*pyrG89; argB2; surG::sgfp::AfpyrG$^+$; nkuAΔ::argB$^+$; pilA::mrfp::AfpyrG$^{+;}$ riboB2*	Vangelatos et al., 2010
AGAS1	*annce102::sgfp::AfpyrG$^+$; argB2; nkuAΔ::argB$^+$; pyroA4; pyrG89*	This work
AGAS2	*annce102::mrfp::AfpyrG$^+$; argB2; nkuAΔ::argB$^+$; pyroA4; pyrG89*	This work
AGAS3	*annce102Δ::AfpyrG$^+$; argB2; nkuAΔ::argB$^+$; pyroA4; pyrG89*	This work

AGAS4	*annce102Δ::Afribo*$^{+}$*; argB2; nkuAΔ::argB*$^{+}$*; pyroA4; riboB2*	This work
AGAS5	*marv1Δ::Afribo*$^{+}$*; argB2; nkuAΔ::argB*$^{+}$*; pyroA4; riboB2*	This work
AGAS6	*marv2Δ::Afpyro*$^{+}$*; argB2; nkuAΔ::argB*$^{+}$*; pyroA4; pyrG89*	This work
AGAS7	*pilA::sgfp::AfpyrG*$^{+}$*; annce102::mrfp::AfpyrG*$^{+}$*; argB2; nkuAΔ::argB*$^{+}$*; pyroA4; pyrG89*	This work
AGAS8	*shrA::sgfp:: pyr4 ; pyrG89*$^{+}$*; pabaB22*	Erpapazoglou et al., 2006
AGAS9	*annce102::mrfp::AfpyrG*$^{+}$*; surG::sgfp::AfpyrG*$^{+}$*; argB2; nkuAΔ::argB*$^{+}$*; pyroA4; pyrG89*	This work
AGAS9	*shrA::sgfp:: pyr4; annce102::mrfp::AfpyrG+; argB2; nkuAΔ::argB*$^{+}$*; pyroA4*	This work
AGAS10	*pilAΔ::Afribo*$^{+}$*; annce102::mrfp::AfpyrG*$^{+}$*; argB2; nkuAΔ::argB*$^{+}$*; pyroA4; pyrG89*	This work
AGAS11	*pilAΔ::Afribo*$^{+}$*; annce102::gfp::AfpyrG*$^{+}$*; argB2; nkuAΔ::argB*$^{+}$*; pyroA4; pyrG89*	This work
AGAS12	*pilAΔ::Afribo*$^{+}$*; annce102::mrfp::AfpyrG*$^{+}$*; surG::sgfp::AfpyrG*$^{+}$*; pyroA4; pyrG89*	This work
AGAS13	*pilAΔ::Afribo*$^{+}$*; annce102Δ::AfpyrG*$^{+}$*; argB2; nkuAΔ::argB*$^{+}$*; pyroA4; pyrG89*	This work
AGAS14	*annce102Δ::AfpyrG*$^{+}$*; surG::sgfp::AfpyrG*$^{+}$*; pilA::mrfp::AfpyrG*$^{+}$*; pyroA4; pyrG89*	This work
AGAS15	*annce102Δ::AfpyrG*$^{+}$*; pilA::sgfp::AfpyrG*$^{+}$*; pyroA4; pyrG89*	This work
AGAS16	*alcA::annce102::mrfp::AfpyrG*$^{+}$*; argB2; nkuAΔ::argB*$^{+}$*; pyroA4; pyrG89*	This work
AGAS17	*alcA::annce102::mrfp::AfpyrG*$^{+}$*; pilA::sgfp::AfpyrG*$^{+}$*; pyroA4; pyrG89*	This work
AGAS18	*pilAΔ::Afribo*$^{+}$*; argB2; nkuAΔ::argB*$^{+}$*; pyroA4*	This work
AGAS19	*annce102Δ::AfpyrG*$^{+}$*; nkuAΔ::argB*$^{+}$ *; pyroA4 ; argB2*	This work

AGAS20	*annce102Δ::AfpyrG*$^{+}$; *pilAΔ::Afribo*$^{+}$; *nkuAΔ::argB*$^{+}$; *pyroA4; argB2*	This work
AGAS21	*niiA::ypkA::AfpyrG*$^{+}$; *nkuAΔ::argB*$^{+}$; *pyroA4; argB2*	This work
AGAS22	*niiA::ypkA::AfpyrG*$^{+}$; *pilAΔ::Afribo*$^{+}$; *argB2; nkuAΔ::argB*$^{+}$; *pyroA4; argB2*	This work
AGAS23	*niiA::ypkA::AfpyrG*$^{+}$; *annce102Δ::AfpyrG*$^{+}$; *nkuAΔ::argB*$^{+}$; *pyroA4; argB2*	This work
AGAS24	*niiA::ypkA::AfpyrG*$^{+}$; *annce102Δ::AfpyrG*$^{+}$; *pilAΔ::Afribo*$^{+}$; *nkuAΔ::argB*$^{+}$; *pyroA4; argB2*	This work
AGAS25	*basA1; nkuAΔ::argB*$^{+}$; *pyroA4; argB2*	This work
AGAS26	*basA1; pilAΔ::Afribo*$^{+}$; *argB2; nkuAΔ::argB*$^{+}$; *pyroA4*	This work
AGAS27	*basA1; annce102Δ::AfpyrG*$^{+}$; *nkuAΔ::argB*$^{+}$; *pyroA4; argB2*	This work
AGAS28	*basA1; annce102Δ::AfpyrG*$^{+}$; *pilAΔ::Afribo*$^{+}$; *nkuAΔ::argB*$^{+}$; *pyroA4; argB2*	This work
AGAS29	*basA1; niiA::ypkA::AfpyrG*$^{+}$; *nkuAΔ::argB*$^{+}$; *pyroA4; argB2*	This work
AGAS30	*marv1Δ::Afribo*$^{+}$; *nkuAΔ::argB*$^{+}$; *pilA::sgfp::AfpyrG*$^{+}$; *pyroA4;*	This work
AGAS31	*marv2Δ::Afpyro*$^{+}$; *nkuAΔ::argB*$^{+}$; *pilA::sgfp::AfpyrG*$^{+}$;	This work
AGAS32	*marv2Δ::Afpyro*$^{+}$; *marv1Δ::Afribo*$^{+}$; *pilA::sgfp::AfpyrG*$^{+}$;	This work
VS85	*pyrG89 pilBΔ::AfpyrG*$^{+}$; *pabaB22; riboB; nkuAΔ::argB*$^{+}$	Vangelatos et al., 2010
AGAS34	*pilBΔ::AfpyrG*$^{+}$; *annce102::mrfp::AfpyrG*$^{+}$; *surG::sgfp::AfpyrG*$^{+}$; *argB2; nkuAΔ::argB*$^{+}$; *pyroA4; pabaB22*	This work
AGAS34	*pilBΔ::AfpyrG*$^{+}$; *annce102::mrfp::AfpyrG*$^{+}$; *surG::sgfp::AfpyrG*$^{+}$; *argB2; nkuAΔ::argB*$^{+}$; *pyroA4; pabaB22; pilAΔ::Afribo*$^{+}$;	

Supplementary Table S2 | Oligonucleotides used in this study

**Name**	**Sequence (5'to 3')**
ANID_07683 1	CGACAAGAATAAGACGCGGGATCTCC
ANID_07683 3	CTCCAGCGCCTGCACCAGCTCCAACCTGCGCCTATGAAGGACGGG
ANID_07683 4	CCCTCAGGGACAAGTCGACCTGTGCACTGCCAACGGTTAAGCTGCTGCG
ANID_07683 6	CGCCGTAGCGCTGCCGTATATG
ANID_07683 p1491 R_GFP pyrG F	CCCGTCCTTCTATGGCGCAGGTTGGAGCTGGTGCAGGCGCTGGAG
ANID_07683 p1491 R_GFP pyrGR	CGCAGCAGCTTAACCGTTGGCAGTGCACAGGTCGACTTGTCCCTGAGGG
ANID_07683 5 Aat II for	CGCGGACGTC GCTATCAGGTAGTCAGTGAAGCAG
ANID_07683 5 Not I rev	CGCGGCGGCCGC GAGATATAGTGATGAGGAGGTGGTC
ANID_07683 3 Not I for	CGCGGCGGCCGC ACTGCCAACGGTTAAGCTGCTGC
ANID_07683 3 Nde I rev	CGCGCATATG GCTGGGCCTGAGTAGGAGCGAAG
AFriboB Not I for	CGCGGCGGCCGC AAGCTTGATATCACAATCAGC
AFriboB Not I REV	CGCGGCGGCCGC CCCGGGCTGCAGGAATTCGATAAG
ANID_07683 2 nest	CTGCAATCTTGCAACCGGCGAAC
ANID_07683 5 nest	GTTGATGGTGAGTCTGCTGGGCC

AN8278 5 KpnI F	CGCGGGTACCGCCGTGTGCGGAGTCATTGAAGG
Nce102-ORF-NcoI	CTCACCATGGGCTTCAGAGGAATCCAGCTC
AN8278 5 XhoI R	CGCGCTCGAGGGGATCCGATACTCGCCGACAAG
AN8278 3 XhoI F	CGCGCTCGAGGATACCTTACGCCATGACGTCATC
AN8278 3 SacII R	CGCGCCGCGGGCTGGGAGATCAGAGGATATCCAG
AN8422DAfpyofor	GGGCTGGGTTACCTCACTATCACGCTCTAGAACTAGTGG ATCC
AN8422DAfpyoRE V	GTTTCCACCAGTGAGCGCTCCCTCGAGGTCGACGGTATC G
AN8422P1	AACCTGGTCGTGGTACGGTC
AN8422P2	TGGGTGCGGTGTGAGTAAGG
AN8422P5	CCGGCGCCTCATTCTAACCC
AN8422P6	GACCACTCAGAGGCCGCATC
AN8422P3	CAGCGCCTGCACCAGCTCCAGCATAACTGACGCCCTTCC C
AN8422P4	GCATCACGCATCAGTGCCTCCCGGGTCAGTCAGGTAGGG TTG
SurG FORWARD RT PCR	CATTCCTTCTGCCTTCATCG
SurG REVERSE RT PCR	TCCTTTCGACCAACAGTTCC

NCE102 RT PCR FORWARD	TCATCTGGACACTGCTCGTC
NCE102 RT PCR REVERSE	ACCTGCGCCATAGAAGGAC
